# ΔAlaAz cleavable linkers: efficient peptide cleavage in the presence of aromatic thiols and air oxygen

**DOI:** 10.1039/d6cb00161k

**Published:** 2026-08-12

**Authors:** Wai-Lun Chan, Alexander A. Vinogradov

**Affiliations:** a Department of Pharmacy and Pharmaceutical Sciences, Faculty of Science, National University of Singapore Singapore 117544 vngrdv@nus.edu.sg

## Abstract

Selective disassembly of multifunctional molecules through the use of cleavable linkers is central to many aspects of chemical biology, biochemistry, and pharmaceutical science. Ideally, cleavable linkers are structurally small and chemically stable on their own but fragment rapidly in the presence of a specific trigger without damaging the rest of the molecule. Here, we report the discovery of the facile oxidative cleavage of 2-(1-amidovinyl)azole-containing peptides (ΔAlaAz; Az: thiazole or oxazole) in the presence of aromatic thiols and air oxygen under ambient conditions. The reaction leads to quantitative peptide cleavage in minutes (<5 min for some substrates) in aqueous buffers at room temperature. We demonstrate that the ΔAlaAz cleavable linkers can be utilized to (i) linearize complex cyclic peptides, (ii) aid the structure determination of ΔAlaAz-containing natural products, and (iii) facilitate the isolation of peptides from cell lysates. Altogether, our work adds an efficient method to the toolbox of cleavable linkers in chemical biology.

## Abstract 2

ΔAlaAz-containing peptides undergo rapid cleavage in the presence of aromatic thiols and air oxygen under ambient conditions. The speed and efficiency of the reaction suggest that ΔAlaAz can be used as cleavable linkers in chemical biology.

## Graphical abstract

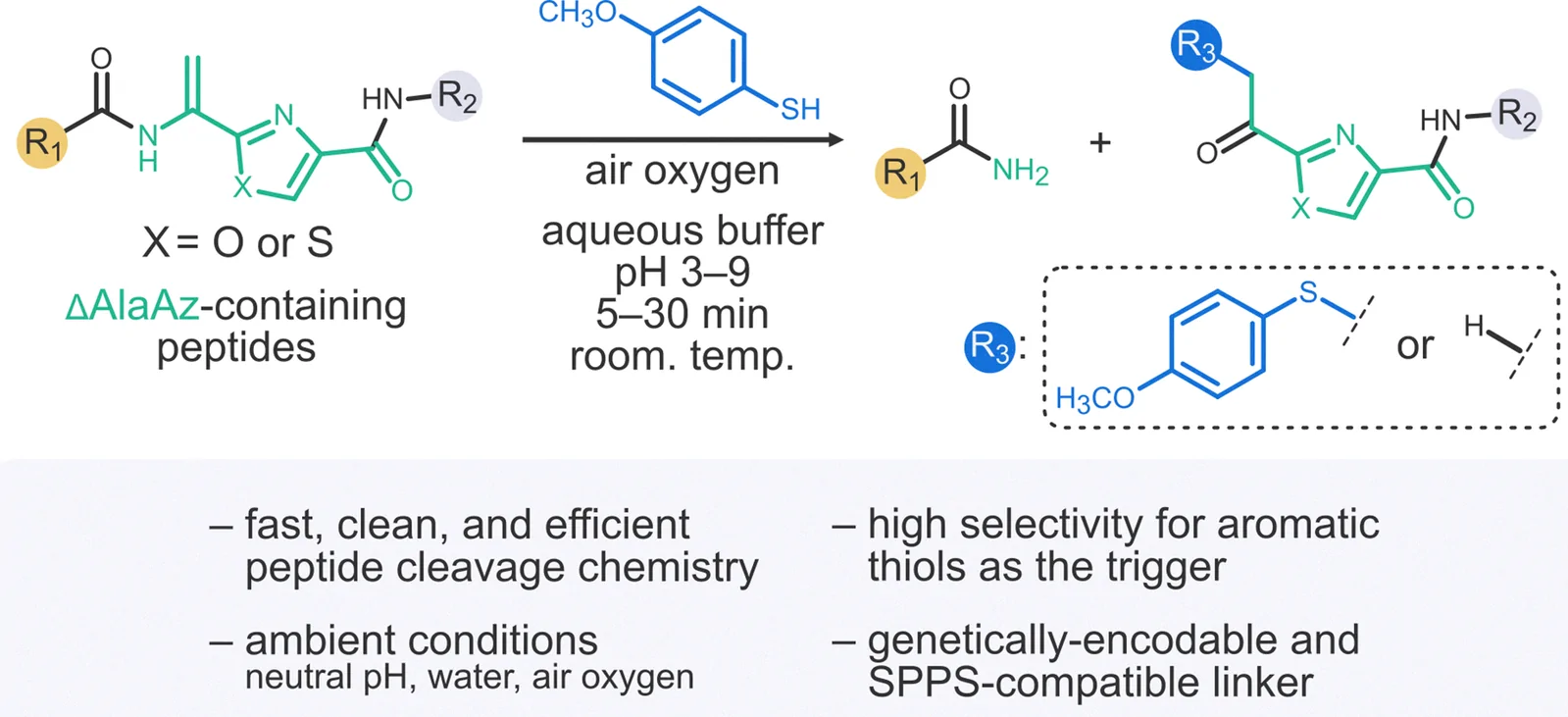

## Introduction

Selective disassembly of multifunctional molecules through the use of cleavable linkers is central to many aspects of chemical biology, biochemistry, and pharmaceutical science.^1–3^ In drug delivery, antibody- or peptide–drug conjugates feature cytotoxic small-molecule payloads conjugated to antibody or peptide homing vehicles *via* a cleavable linker, which can be selectively immolated upon reaching the site of delivery to release the biologically active drug.^4^ In chemical biology and proteomics, cleavable linkers can be used for the efficient pulldown of compounds of interest.^5–8^ Capture probes often contain a moiety which enables the release of captured compounds with an orthogonal chemical trigger. The orthogonality of the trigger ensures the clean recovery of the species of interest from complex biological samples such as cell lysates and tissue homogenates.^6,9^ Analogously, cleavable linkers are often employed in combinatorial peptide chemistry during affinity selection experiments or to linearize cyclic peptides for *de novo* sequencing by mass spectrometry.^10–13^

When it comes to peptides and proteins, dozens of efficient cleavage methods have been devised over the years. Among the most popular strategies are the designs responsive to thiols,^14–17^ enzymes,^18–20^ oxidation or reduction,^7,21–28^ acids or bases,^20,29,30^ and photochemical triggers (Fig. 1a).^31–33^ A good cleavable linker design is based on the chemical structures that are sufficiently stable on their own but undergo fast and clean fragmentation upon encountering a mild trigger, with minimal damage to the rest of the molecule. For example, antibody–drug conjugates often employ the cleavable linkers that are metabolically and chemically stable in the biological milieu, but can be rapidly and efficiently cleaved by lysosomal enzymes.^4^ Ideal cleavable linkers are also structurally small and easy to install.

**Fig. 1:**
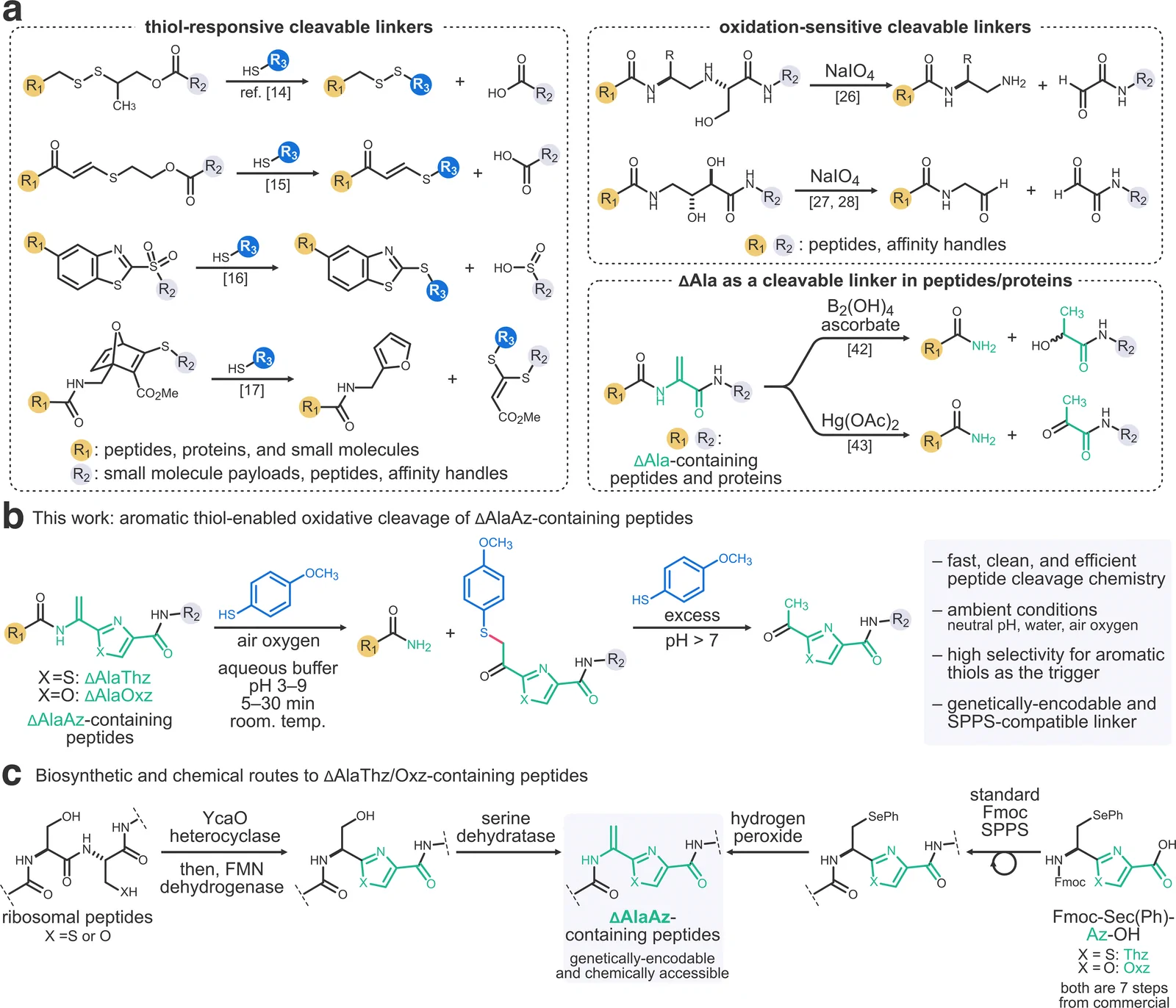
Cleavable linkers in chemical biology. (a) An overview of representative cleavable linker designs featuring thiol-responsive and oxidation-sensitive strategies, as well as the ΔAla-based approaches. (b) Aryl thiol-mediated oxidative cleavage of ΔAlaAz-containing peptides reported in this work. (c) ΔAlaAz-containing peptides can be accessed from Ser–Ser- and Ser–Cys-containing ribosomal substrates in a three-step enzymatic cascade (the left pathway); in this way, ΔAlaAz-containing peptides are genetically encodable. The same structures can also be furnished synthetically from appropriate selenocysteine derivatives (the right pathway). SPPS: solid-phase peptide synthesis.

We are broadly interested in studying the reactivity of α,β-dehydroamino acid (dhAA)-containing peptides and proteins.^34^ dhAAs are non-proteinogenic amino acids usually accessed *via* post-translational (or post-synthetic) modification of polypeptides. In the proteome, dhAAs such as dehydroalanine (ΔAla) can spontaneously form *via* a non-enzymatic elimination of phosphate from phospho-Ser, and in this way, affect protein function.^35,36^ dhAAs are also found in the structures of many peptidic natural products and *de novo* discovered biologically active peptides.^37,38^ Because dhAAs are electrophilic to varying degrees, they can serve as reactive warheads for the covalent conjugation of peptide ligands to target proteins upon binding.^39–41^ Additionally, ΔAla can play the role of a cleavable linker as it can be selectively fragmented *via* hydrolytic or reductive pathways (Fig. 1a).^42,43^

In the course of our investigations into the reactivity of various dhAAs (to be reported separately), we found that 2-(1-amidovinyl)thiazole- (ΔAlaThz) or analogous oxazole-containing peptides (ΔAlaOxz; collectively referred to as ΔAlaAz) undergo facile oxidative cleavage in the presence of suitable aromatic thiols (Fig. 1b). In this paper, we report the initial discovery, mechanistic investigations, and potential applications of this unusual chemistry. We find that the cleavage likely involves a radical pathway and utilizes air oxygen. The reaction is remarkable in that quantitative cleavage to well-defined products can be achieved in minutes (<5 min for ΔAlaThz) under ambient conditions – in aqueous buffer at room temperature, and in the presence of an aromatic thiol and air oxygen. We demonstrate that the ΔAlaAz cleavable linkers can be utilized to (i) linearize complex cyclic peptides, (ii) aid the structure determination of ΔAlaAz-containing natural products, and (iii) facilitate the isolation of peptides from cell lysates. Altogether, our work adds an efficient method to the toolbox of cleavable linkers in chemical biology.

## Results and discussion

### Initial reaction discovery

ΔAlaAz dhAAs are found natively in several ribosomally synthesized and post-translationally modified peptides, notably in the thiopeptide family^44,45^ of natural products (lactazole,^46^ berninamycin,^47^ sulfomycin,^48^ cyclothiazomycin)^49^ and in some azole-containing peptides (goadsporin^50^ and carnazolamide).^51^ These structures are accessed from Ser–C̲y̲s̲- or Ser–S̲e̲r̲-containing peptides in a three-step enzymatic pathway that begins with cyclodehydration and dehydrogenation of the underlined Cys or Ser residues (Fig. 1c).^52^ The resulting azole-containing dipeptides are then dehydrated at the remaining Ser to furnish the final ΔAlaAz moieties.^53^ The enzymes responsible for this process are often highly promiscuous and can accept a variety of structurally diverse substrates to install ΔAlaAz motifs in non-native ribosomal peptides.^54–57^ In this way, ΔAlaAz linkers are in principle genetically encodable. The same structures can also be accessed by solid-phase peptide synthesis from suitable selenocysteine (Sec) derivatives after post-synthetic oxidative elimination.^58–60^

Here, we opted to work with synthetic constructs and prepared three dhAA-containing model peptides, pep.01–03, which differed only in the nature of the central dhAA (ΔAla, ΔAlaOxz, and ΔAlaThz; Fig. 2a), to study their reaction with various thiols (Fig. 2b; SI 2.1 and 2.2). As expected, all three peptides cleanly reacted with aliphatic thiols such as cysteamine *via* a thia-Michael conjugate addition^34^ to yield two diastereomeric products according to LC/MS analysis. ΔAlaAz were considerably less electrophilic than ΔAla; whereas the latter underwent complete conjugation in 4 h at pH 7.4 and 37 °C, less than 35% conversion was observed for ΔAlaThz and ΔAlaOxz.

**Fig. 2:**
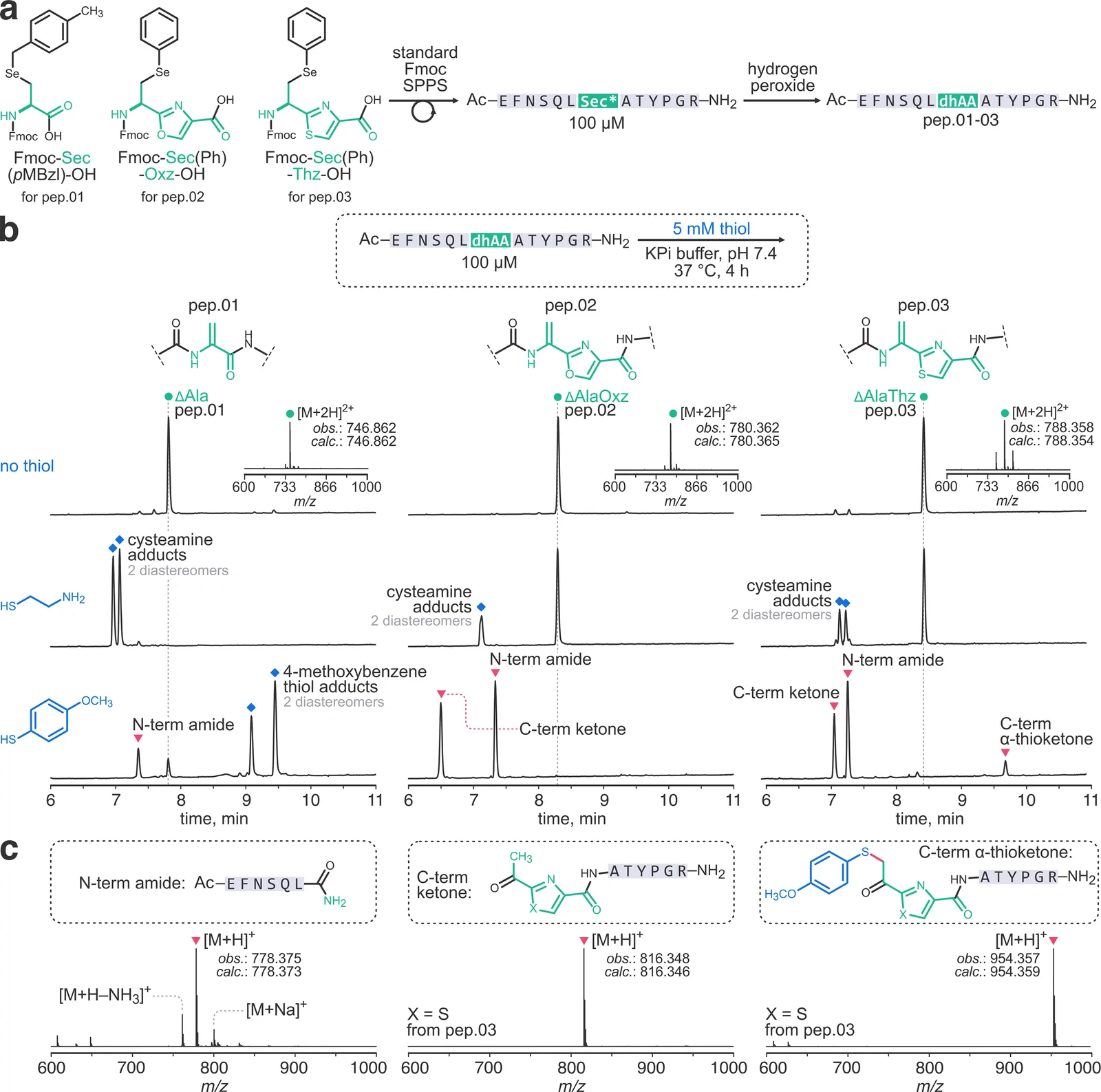
Initial reaction discovery. (a) The general synthetic strategy used to prepare the dhAA-containing peptides pep.01–03. Synthetic schemes for the Sec amino acids are shown in Fig. S10. (b) Peptides pep.01–03 were incubated with aliphatic or aromatic thiols as indicated in the scheme. See SI 2.1 and 2.2 for reaction protocol details. Displayed are the annotated total ion current (TIC) LC/MS chromatograms showing reaction outcomes; ESI-MS mass spectra for the starting peptides are shown as insets. (c) Assigned chemical structures for the major reaction products and the associated ESI-MS mass spectra. MS/MS spectra for the products in panel (b) are shown in Fig. S1.

In contrast, when repeating the same reaction with *para*-methoxybenzenethiol (pMBT), neither ΔAlaOxz nor ΔAlaThz gave detectable conjugation products (Fig. 2b). Instead, we observed full conversion of the corresponding peptides to two (for pep.02, ΔAlaOxz) or three (pep.03, ΔAlaThz) new products which were about half the mass of the original compounds, suggesting peptide cleavage. The reaction between ΔAla (pep.01) and pMBT predominantly gave the expected thia-Michael addition products, but was also accompanied by a small amount (<15%) of peptide cleavage. One of the products was identical for all three reactions (Fig. 2c). Based on tandem mass spectrometry (MS/MS; Fig. S1a), we identified it as the C-terminal carboxamide derived from the N-terminal portion of the peptide (referred to hereafter as the “N-term amide”). The same N-term amide formed upon the hydrolytic cleavage of pep.01 at ΔAla, which is known to produce carboxamides,^43^ further supporting this assignment.

The other two products were derived from the C-terminal portion of pep.02 or pep.03; based on MS/MS (Fig. S1b and c) and a number of additional experiments (*vide infra*), they were tentatively assigned as the structures bearing either 2-acetylazole (C-term ketone) or the same moiety, but α-thio-substituted (C-term α-thioketone; Fig. 2c). The formation of C-term α-thioketones indicated a redox process rather than simple hydrolysis, because α-thioketones formally exist in a higher oxidation state than the original ΔAlaAz structures.

### Reaction mechanism investigations

To investigate this reaction further, we first incubated pep.02 and pep.03 with pMBT in buffers of various pH (3.6, 5.2, 7.4, and 8.6; Fig. 3a and S2a) at 37 °C for 4 h. Under mildly acidic conditions (pH 3.6 and 5.2), the peptides were converted into the corresponding N-term amide and C-term α-thioketone products. At higher pH (7.4 and 8.6), the ketones formed instead of the α-thioketones as the primary C-terminal products. Quantitative cleavage was observed under all tested conditions, which argues against the involvement of the thiolate anion in the reaction, and by extension, against a thia-Michael-type process.

**Fig. 3:**
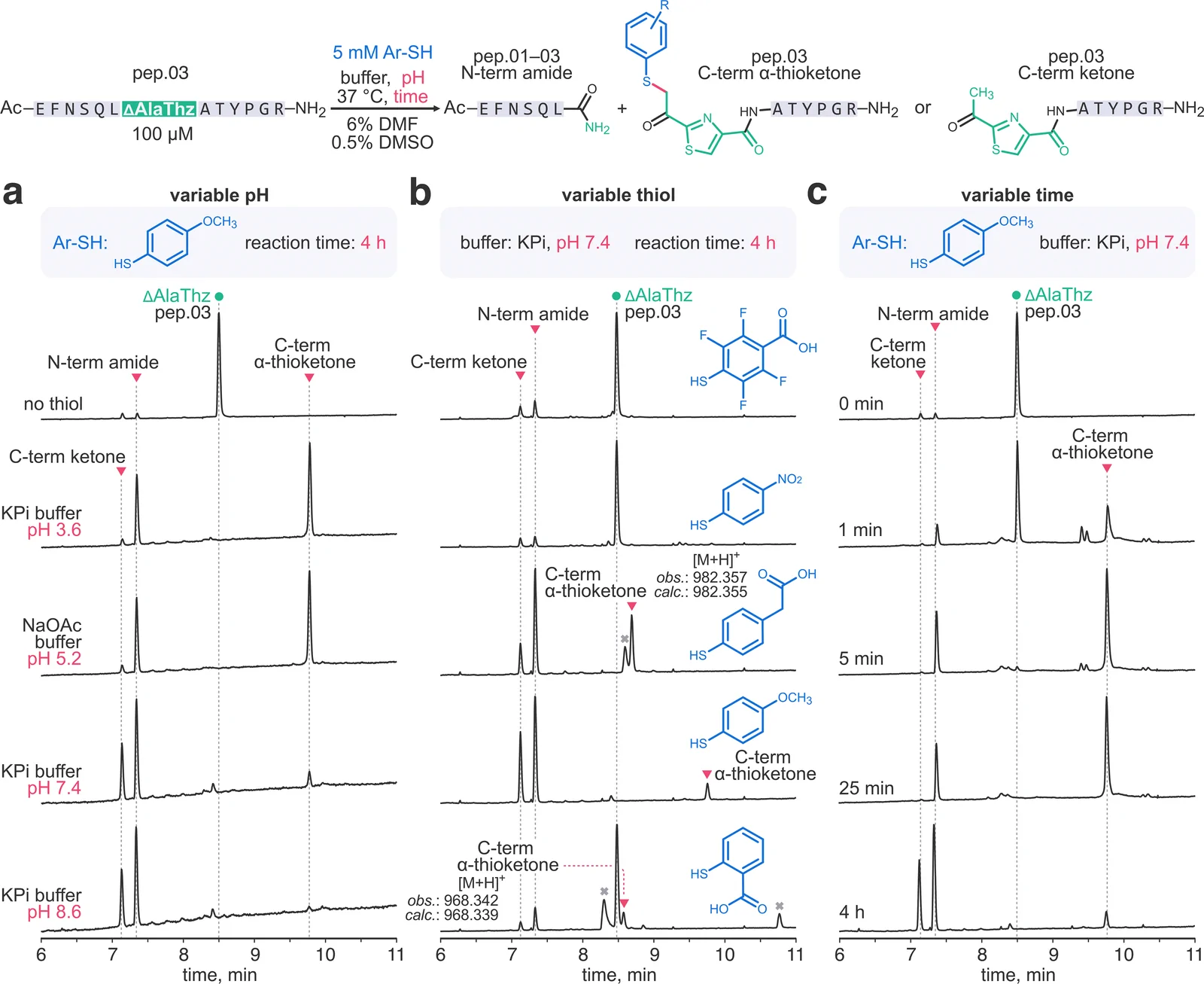
Initial reaction mechanism investigations. Peptide pep.03 was incubated with aryl thiols as indicated in the scheme and the reaction outcomes were analyzed by LC/MS. See SI 2.1 for reaction protocol details. In each case, displayed are the annotated TIC LC/MS chromatograms showing reaction outcomes for the pH (panel (a)), aryl thiol (b) and time course (c) studies. Peaks labelled with a grey cross (×) were small-molecule contaminants. Analogous data for pep.02 is shown in Fig. S2.

In further experiments, we investigated whether the nature of the aromatic thiol affected reaction outcomes and found that in addition to pMBT (p*K*_a_ = 6.8),^61^ the use of 4-mercaptophenylacetic acid (6.6)^62^ led to the quantitative cleavage of pep.02 and 03 (Fig. 3b and S2b). In contrast, 4-nitrobenzene thiol (4.7),^61^ tetrafluoro-4-mercaptobenzoic acid (<3.0; the exact value is unknown), and thiosalicylic acid (7.8)^63^ were marginally effective. In other words, there was no monotonic relationship between thiol p*K*_a_ and cleavage reaction efficiency which again argues against the process based on a thia-Michael conjugate addition.

A time course experiment conducted for pep.02 and pep.03 using pMBT at pH 7.4 showed that the cleavage reaction was fast, particularly for pep.03 (ΔAlaThz; Fig. 3c). For pep.03, the cleavage products were observed after 1 min, and the starting material was completely consumed after a 5 min reaction. Importantly, this experiment indicated that the C-term α-thioketone is an intermediate *en route* to the C-term ketone. After 5 min, the reaction mixture contained the α-thioketone as the primary C-terminal product, but upon longer incubation, it gradually transformed to the ketone. A similar experiment with pep.02 led to the same conclusions (Fig. S2c); in this case, the ketone was the only C-terminal product after a 4 h reaction. Taken together, these results strongly suggest a redox cleavage reaction, in which the N-term amide and the C-term α-thioketone form as the initial products. Then, at a sufficiently high pH (pH 7.4 or 8.6), the latter intermediate can be reduced to the C-term ketone. At low pH (pH ≤ 5.2), the C-term α-thioketone appeared stable.

Next, we attempted to disrupt the cleavage and intercept reaction intermediates. Although dhAAs are primarily known to react as electrophiles similarly to acrylamides, structurally, they are also enamides, and there are several examples where their reactivity as enamides is manifested.^64–66^ We first hypothesized that aromatic thiols may be oxidized by air oxygen to transient sulfenic acids, which are strongly electrophilic and may react with ΔAlaAz in a process that could explain the formation of the C-term α-thioketones (Fig. S3). However, a reaction between pep.02 or pep.03 and 5 mM pMBT at pH 3.6 for 15 min in the presence of excess dimedone (10 mM) still led to quantitative cleavage (Fig. 4a and S4). Dimedone is known to react with sulfenic acids to furnish thioethers with rate constants exceeding 10 M^−1^ s^−1^.^67^ Accordingly, it is often used as a chemical biology probe for sulfenic acids and has also been employed to intercept sulfenic acid-based pathways.^67,68^ Its ineffectiveness in preventing cleavage argues against the involvement of sulfenic acid intermediates.

**Fig. 4:**
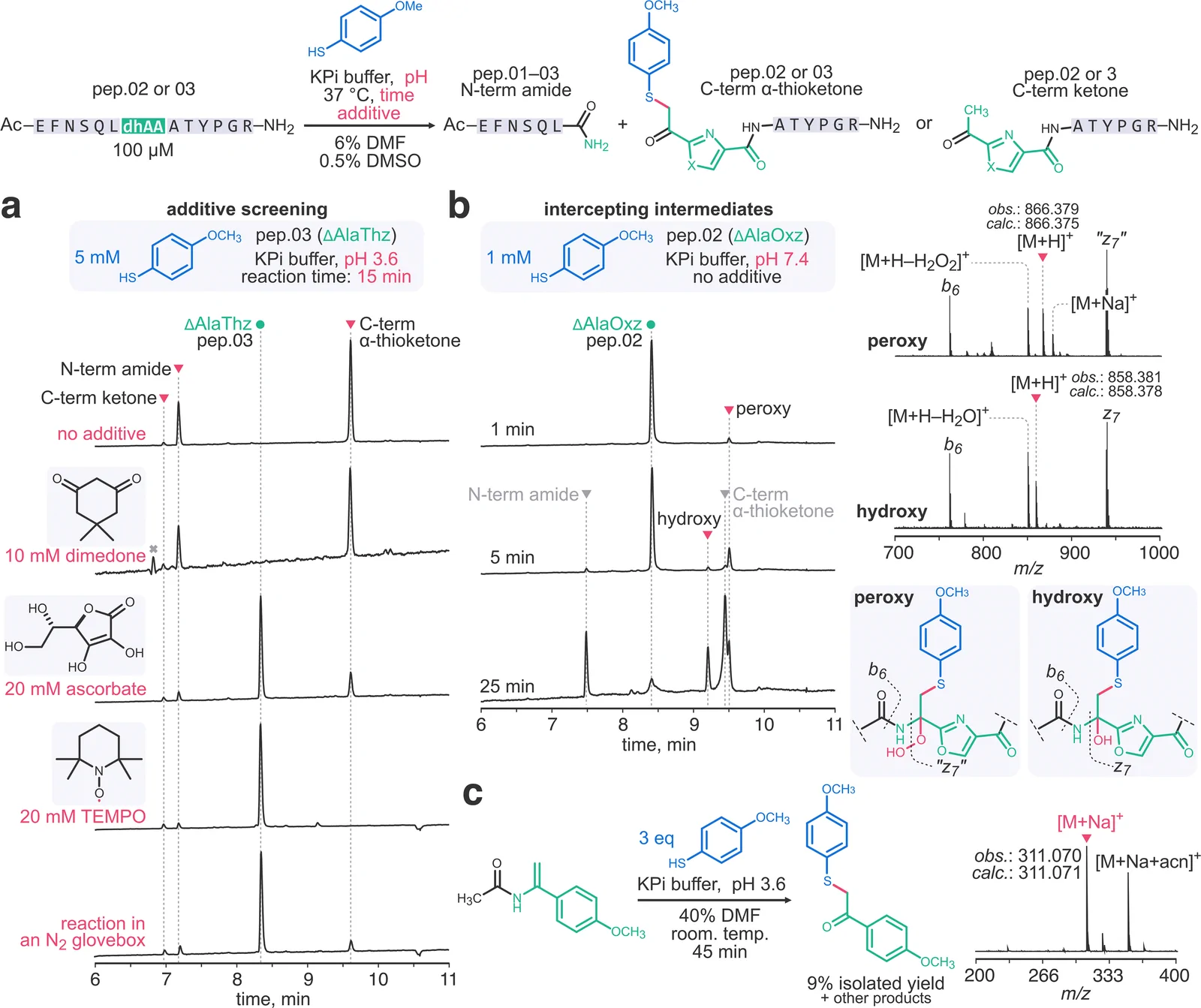
Additional reaction mechanism investigations. Peptides pep.02 or pep.03 were incubated with pMBT as indicated in the scheme and the reaction outcomes were analyzed by LC/MS. In each case, displayed are the annotated TIC LC/MS chromatograms showing reaction outcomes. See SI 2.1 for reaction protocol details. (a) The additive screening study. Peaks labelled with a grey cross (×) were small-molecule contaminants. Analogous data for pep.02 is shown in Fig. S4. (b) TIC chromatograms for the intermediate interception experiments. Also shown are the chemical structures assigned to the two major intermediates and the associated annotated ESI-MS mass spectra. (c) A reaction between a small molecule mimic of ΔAlaAz-containing peptides and pMBT results in formation of the α-thioketone. See additional details in SI 2.4.

In contrast, reactions in the presence of 20 mM sodium ascorbate or 20 mM TEMPO either substantially suppressed (ascorbate) or completely abolished (TEMPO) the cleavage of pep.02 and pep.03 (Fig. 4a and S4). Ascorbate and TEMPO are efficient interceptors of radical intermediates,^69,70^ which strongly suggested that the cleavage involves a radical process. Further, running the reaction in a nitrogen glovebox also led to the minimal cleavage of pep.02 and pep.03 pointing to the involvement of oxygen. For all reactions outside a glovebox, no particular care was taken to introduce oxygen; no air bubbling, oxygen layering, or stirring was performed, which – given the reaction rate – suggests high sensitivity of the system to oxygen. The presence of trace transition metals, particularly Fe^2+^ and Cu^2+^, can promote thiyl radical formation through a Fenton-like chemistry.^71,72^ However, reactions conducted with pep.02 in the presence of 20 mM sodium cyanide or 20 mM ethylenediaminetetraacetic acid, both of which inhibit thiol oxidation,^73,74^ resulted in quantitative cleavage within 15 min, suggesting that metal catalysis is unlikely to play a major role in the process (Fig. S5a).

In the aforementioned time course experiment, in addition to the discussed products, several unidentified intermediates were observed by LC/MS after short incubation times (1–5 min). They eventually transformed to the N-term amide and C-term α-thioketones. We intercepted these intermediates when running reactions between pep.02 and 1 mM pMBT at pH 7.4 for 1–25 min and quenching them with *N*-methylmaleimide to consume the excess thiol (Fig. 4b). LC/MS analysis showed clear accumulation of two intermediates derived from the full-length peptide. Both compounds were fragile and underwent significant fragmentation in ESI-MS. Based on their mass, fragmentation patterns, as well as the known reactivity of dhAAs in radical processes in the presence of oxygen,^75^ we assigned one intermediate as the α-peroxy-peptide (peroxy), and the other as the α-hydroxy-peptide (hydroxy; Fig. 4b).^76,77^ The time course analysis indicated that the peroxy species forms first and is then converted to the α-hydroxy-peptide, which decomposes to give the N-term amide and the C-term α-thioketone. However, the possibility that the peroxy intermediate can additionally undergo direct cleavage to the C-term α-thioketone can not be excluded.

Finally, to substantiate the structural assignment of the key C-term α-thioketone, we prepared *N*-(1-(4-methoxyphenyl)vinyl)acetamide as a small molecule mimic of ΔAlaAz moieties, and subjected it to reaction with 3 eq. of pMBT at room temperature and pH 3.6 for 45 min (Fig. 4c). The reaction led to complete consumption of the starting enamide and resulted in the formation of the corresponding α-thioketone, which was isolated and characterized alongside other reaction products (SI 2.4). These results reinforce our structural assignments of peptide cleavage products.

Altogether, our data suggests a plausible reaction mechanism as indicated in Fig. 5. Upon reaction with oxygen in ambient air, aryl thiols undergo initial oxidation to form relatively stable Ar–S˙ radicals.^78^ It is possible that the radical initiation is aided by light, but the reactions conducted in the dark still led to robust peptide cleavage, suggesting that oxygen involvement is more likely. The Ar–S˙ radical then undergoes addition to the double bond in ΔAlaAz producing the peptide α-radical. This relatively stable species reacts with oxygen, forming the α-peroxy intermediate upon reduction of the initially formed peroxyl radical. Similar peroxy intermediates are a known nuisance in radical additions to ΔAla-containing peptides and proteins, often necessitating glovebox conditions.^75,79–81^ The peroxy compound is likely reduced by the excess thiol to the α-hydroxy species, which is hydrolytically labile as an *N*-acyl hemiaminal. This intermediate collapses producing the N-term amide and C-term α-thioketone cleavage products. At a sufficiently high pH, the latter compound can be further reduced to the corresponding ketone. We do not have direct evidence for how this process takes place, but facile reduction of α-thioketones is well known. It can happen photochemically,^82,83^ electrochemically,^84^ thermally,^85^ and in the presence of single-electron reducing agents.^86^ We hypothesize that the aromatic thiolate abundant at a pH above thiol's p*K*_a_ acts as a reducing agent through single-electron transfer either directly or after reversible association to a thiyl radical to form a strongly reducing disulfide radical anion.^87,88^ The reduction leads to the scission of the S–C bond and formation of the ketone after tautomerization.^89^

**Fig. 5:**
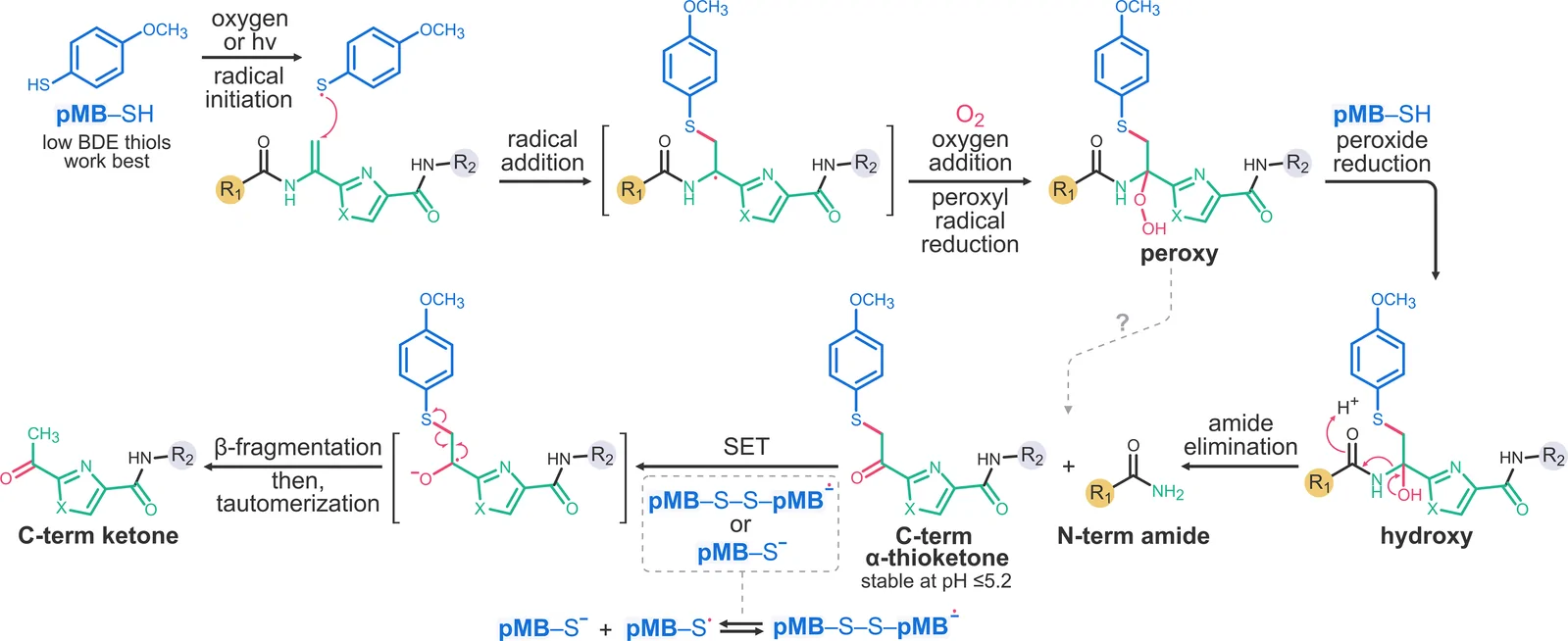
Proposed reaction mechanism. See the accompanying text for discussion.

This is a remarkable sequence of mechanistic steps because it all happens spontaneously under ambient conditions, in the absence of dedicated radical initiators, and without deliberate exposure of the reaction mixtures to oxygen. With the exception of the α-thioketone reduction, the reaction rate is also notable, particularly for ΔAlaThz, as quantitative peptide cleavage is achieved in *ca.* 5 min. The proposed mechanism suggests an autocatalytic process, because additional radical species are generated as the reaction progresses, and our limited time course data does suggest that cleavage accelerates gradually over time.

### ΔAlaAz as cleavable linkers

In the final series of experiments we aimed to demonstrate the utility of the discovered chemistry and show its generality beyond model peptides. First, we showed that pMBT-mediated cleavage can linearize cyclic ΔAlaThz-containing peptides. Peptide linearization is an important strategy employed in synthetic combinatorial chemistry.^10–13^ Affinity selection from combinatorial libraries of cyclic peptides yields biologically active hits which often must be linearized for MS/MS structure decoding, because MS/MS-based sequencing of cyclic forms can be difficult and impractical. Peptides TP15 and hTP15 are *de novo* discovered natural product-like thiopeptides acting as potent and selective inhibitors of TNIK kinase (Fig. 6a and S6a).^90–92^ Both peptides feature cyclic structures containing an ΔAlaThz-like moiety. The incubation of TP15 or hTP15 with 5 mM pMBT at room temperature and pH 7.4 for 15 min led to nearly quantitative cleavage along the C–N bond in ΔAlaThz, producing the linearized α-thioketone-containing peptide as expected in both cases (Fig. 6a and S6a). For hTP15, the cleavage site was additionally confirmed by MS/MS (Fig. S6b; linearized TP15 did not undergo fragmentation in MS/MS). These experiment demonstrate the feasibility of using our chemistry for peptide linearization.

**Fig. 6:**
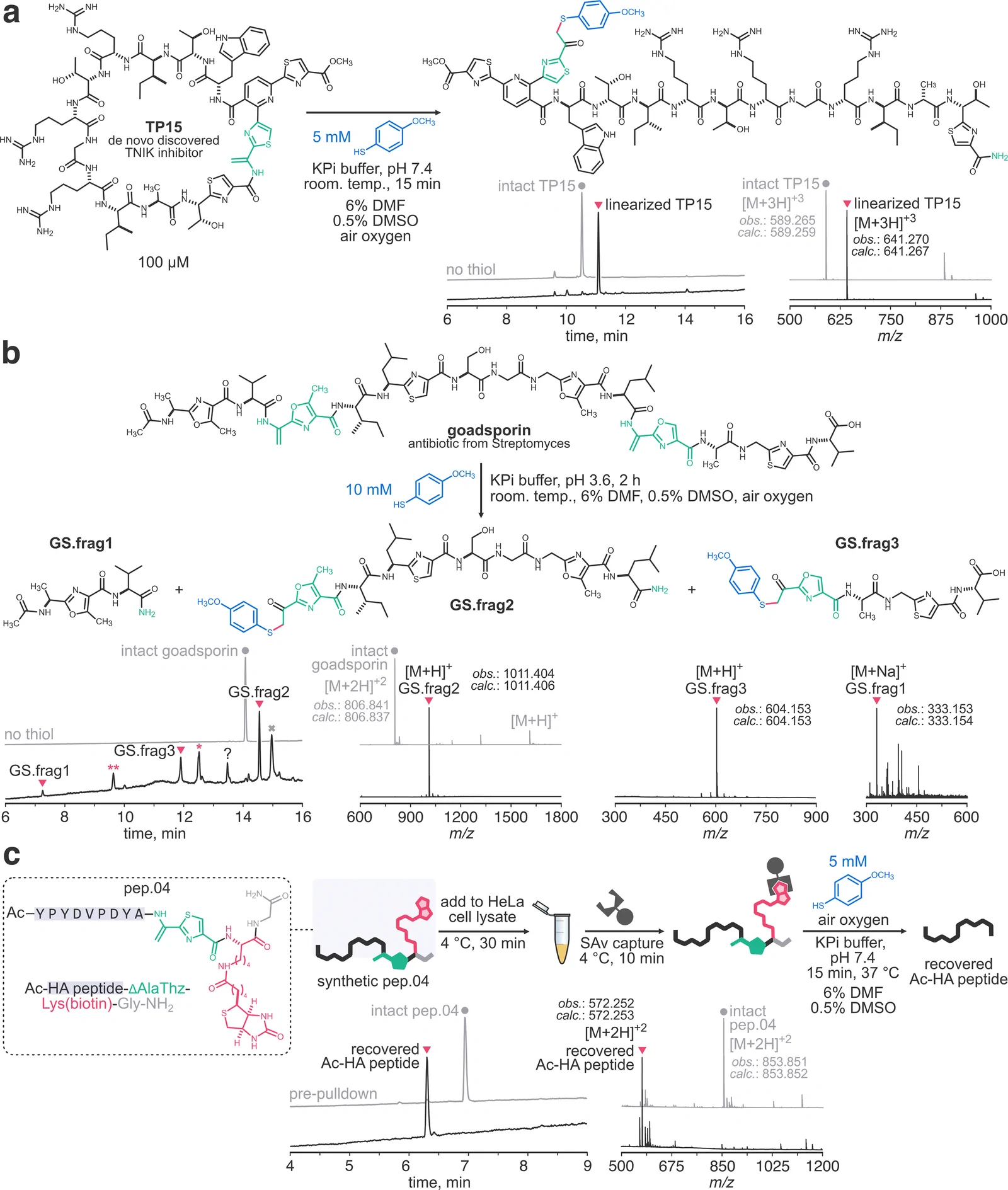
Applications of the discovered cleavage reaction. (a) Linearization of thiopeptide TP15 with pMBT. The reaction was performed as indicated in the scheme. See SI 2.1 for the detailed reaction protocol. Displayed are the annotated TIC LC/MS chromatograms showing the reaction outcomes in the presence (black) or absence (grey) of pMBT. The associated ESI-MS spectra for the biggest chromatogram peak are shown on the right. Analogous data for hTP15 is shown in Fig. S6. (b) Fragmentation of goadsporin with pMBT. The reaction was performed as indicated in the scheme. On the left, displayed are the annotated TIC LC/MS chromatograms showing the reaction outcomes in the presence (black) or absence (grey) of pMBT. *: oxidized GS.frag2 (+16 Da w.r.t. GS.frag2; likely, a sulfoxide); **: analogous oxidized GS.frag3; ?: unknown product derived from goadsporin. Peaks labelled with a grey cross (×) indicate small-molecule contaminants. The ESI-MS spectra for the cleavage products, GS.frag1, GS.frag2, and GS.frag3, are shown on the right. MS/MS spectra for GS.frag2 and GS.frag3 are provided in Fig. S7. (c) Recovery of Ac-HA peptide from HeLa cell lysate. Displayed are the chemical structure of pep.04 and the schematic description of the lysate recovery experiment. For full experimental details, see SI 2.3. Also displayed are the annotated TIC LC/MS chromatograms showing the recovered Ac-HA peptide (in black) overlaid with the starting pep.04 (grey). The associated ESI-MS spectra for the biggest chromatogram peak are shown on the right. MS/MS spectra for the recovered Ac-HA peptide are shown in Fig. S8.

Because pMBT-mediated peptide cleavage proceeds under mild conditions where most other functional groups remain intact, we propose that it can be used as a test to ascertain the presence of ΔAlaAz motifs in natural products during the structure elucidation process. As a proof-of-concept, we performed the cleavage of goadsporin, a narrow-spectrum natural product antibiotic isolated by from *Streptomyces* sp. TP-A0584 (Fig. 6b).^50^ Structurally, goadsporin is a linear azole-containing peptide containing ΔAlaOxz and ΔAlaOxz^5Me^ amino acids. Quantitative cleavage of goadsporin at pH 3.6 required a longer 2 h incubation with 10 mM pMBT, but still resulted in the formation of three expected products. The identity of the two α-thioketones was additionally confirmed by MS/MS (Fig. S7).

Finally, the mild conditions of the cleavage reaction suggest that it may be useful for affinity pulldowns. To demonstrate this, we prepared pep.04, a peptide containing the N-terminal HA-tag sequence followed by the ΔAlaThz linker and the Lys(biotin) affinity handle (Fig. 6c). We sought to demonstrate that this peptide can be selectively recovered from complex biological samples such as cell lysates using streptavidin-immobilized beads and then released from the beads by pMBT. In the event, pep.04 (20 µM) was incubated with the HeLa cell lysate for 30 min, and then streptavidin-coated dynabeads were added. After a further 10 min incubation, the beads were extensively washed with a urea-containing buffer, and the cleavage solution containing 5 mM pMBT in sodium phosphate buffer at pH 7.4 was added to the suspension. The cleavage reaction was allowed to proceed for 15 min at 37 °C, after which the supernatant was collected. LC/MS analysis showed clean recovery of the HA-tag carboxamide as the single observed compound; no cell lysate components were detected (Fig. 6c and S8). The recovered compound was identical to the N-term amide product observed when running the cleavage reaction with pep.04 in solution (Fig. S9). These results confirm that our cleavage chemistry can be utilized for chemical biology applications to cleanly recover compounds of interest from complex biological mixtures.

## Conclusions

We report the discovery of an efficient cleavage chemistry for ΔAlaAz-containing peptides. High reaction rates, mild conditions, and selectivity for a trigger that is otherwise rarely found in biological systems (aromatic thiols) suggests multiple applications of ΔAlaAz motifs as cleavable linkers in chemical biology; some of these applications are exemplified in the experiments above. The fact that ΔAlaAz structures are reasonably stable, synthetically accessible, and are in principle genetically encodable further adds to the appeal of our strategy. The reactions of ΔAlaThz are particularly rapid and are complete within 5 min. On the other hand, ΔAlaOxz is more chemically stable (to be reported elsewhere).

Our mechanistic investigations – while still in progress – suggest a facile radical process that proceeds without a dedicated radical initiator. At present, it remains unclear what structural features in ΔAlaAz endow them with such sensitivity to Ar–S˙ radicals and/or oxygen. We hope to elucidate such questions, understand whether the reaction can be accelerated further, and study the reactivity of related systems in future work.

## Conflicts of interest

The authors declare no competing interests.

## Supplementary Material

CB-007-D6CB00161K-s001

## Data Availability

Experimental procedures and additional results are summarized in supplementary information (SI). Supplementary information is available. See DOI: https://doi.org/10.1039/d6cb00161k. Other data are available from the corresponding author upon reasonable request.
