# Supplementary material for: ΔAlaAz cleavable linkers: efficient peptide cleavage in the presence of aromatic thiols and air oxygen
Source: RSC Chem Biol. 2026 Aug 12;7(10):2028–39. doi: 10.1039/d6cb00161k (PMC13480372; doi:10.1039/d6cb00161k)

Supplementary Information (SI) for RSC Chemical Biology.
This journal is © The Royal Society of Chemistry 2026

Supporting information for

ΔAlaAz cleavable linkers: efficient peptide cleavage in the presence of aromatic thiols and air oxygen

Wai-Lun Chan[1] and Alexander A. Vinogradov[1,*]

[1] Department of Pharmacy and Pharmaceutical Sciences, Faculty of Science, National University of Singapore, Singapore 117544

* Correspondence to vngrdv@nus.edu.sg

The authors declare no competing interests.

Contents

1. General.. 3
1.1. Materials 3
1.2. Instrumentation 3
1.2.1. LC/MS and MS/MS analysis.. 3
2. Methods.. 4
2.1. pMBT-mediated cleavage of ΔAlaAz-containing peptides... 4
2.2. Thia-Michael conjugation of dhAA-containing peptides to cysteamine........................... 5
2.3. Cell lysate recovery of Ac-HA peptide 5
2.4. Small molecule reactions... 6
2.5. Peptide synthesis 8
3. Supplementary Figures.. 9
4. References 18
5. Characterization data.. 20
5.1. NMR spectra 20
5.2. HPLC/MS Peptide characterization 24

1. General

1.1. Materials

All commercially available materials were used without further purification. Unless otherwise noted, reagents and solvents were purchased from Nacalai Tesque, Wako Pure Chemical Industries, Sigma-Aldrich, BLD Pharmatech, or TCI Chemicals. Goadsporin was a generous gift from Prof. Hiroyasu Onaka (Gakushuin University); thiopeptides TP15 and hTP15 were provided by Dr. Yue Zhang (The University of Tokyo). 4-Methoxybenzenethiol (**pMBT**) was purchased from TCI Chemicals or BLD Pharmatech; the two sources gave identical results.

1.2. Instrumentation

NMR spectra were recorded on a JEOL JNM-ECS400 spectrometer (^{1}H NMR at 400 MHz and ^{13}C NMR at 101 MHz). Chemical shifts for ^{1}H NMR are reported in parts per million (δ) relative to tetramethyl silane in a deuterated solvent as the internal standard. Chemical shifts for ^{13}C NMR are reported in parts per million (δ) relative to the solvent (e.g., $CDCl_3$, δ = 77.16). The following abbreviations are used for spin multiplicity: s = singlet, d = doublet, t = triplet, q = quadruplet, m = multiplet.

High-resolution mass spectra for small molecules were acquired on a Bruker compact QTOF-MS mass spectrometer.

Normal phase column chromatography was performed on Biotage Isolera™ Spektra flash purification system equipped with appropriately sized Biotage SNAP Ultra columns. Reverse phase (RP) preparative HPLC separations utilized a mobile phase consisting of 0.1% trifluoroacetic acid (TFA) (*v/v*) in acetonitrile (Solvent B) and 0.1% TFA (*v/v*) in water (Solvent A) and were performed on a Prominence HPLC (Shimadzu) equipped with an Agilent ZORBAX 300SB-18 PrepHT column (21.2 × 250 mm; 7 μm particle size; 300 Å pore size) at a flow rate of 20 mL/min.

1.2.1. LC/MS and MS/MS analysis

Reaction outcomes were analyzed using Waters Xevo G2-XS QTof instrument equipped with Acquity I-Class UPLC system. HPLC was done on an Acquity UPLC Peptide BEH C18 column [dimensions: 150 x 2.1 mm; pore size: 300Å ; particle size: 1.7 μm] using 0.1% (v/v) formic acid (FA) in water (solvent A') and 0.1% (v/v) FA in acetonitrile (solvent B') as a mobile phase. Analysis was performed at 60 °C and 300 μL/min flow rate. MS analysis was done in a positive polarity/high sensitivity mode with a 0.3 s or 0.5 s scan time. Capillary voltage was set to 700 V; ESI source and desolvation temperatures were 120 and 400 °C, respectively. Manufacturer-supplied Leu-enkephalin was used as a lockspray standard for continuous mass axis referencing, and the lockspray setup procedure was performed according to the manufacturer's instructions prior to every run.

For tandem mass spectrometry, a data-dependent acquisition method was used. Collision-induced dissociation (**CID**) fragmentation was triggered in real time if detected ion intensity exceeded $5 \cdot 10^4$ and z = 1 or 2. MS/MS spectra were acquired with a 2 s scan time and parameterized collision energy values. In method 1, collision energies were set to ramp from 6–8 to 15–22 eV over the range of acquired *m/z* values (200 to 2000); in method 2, these values were 6–8 to 22–28 eV, and in method 3, 6–8 to 30–40 eV. Samples subject to MS/MS were reanalyzed three times to acquire MS/MS spectra for each method. All acquired spectra were analyzed, but for brevity, reported are only the most informative ones.

LC-MS data was analyzed with MassLynx v.4.1. MS/MS assignments were done manually. For a given peptide, a series of possible structures were generated, and for each of these structures, ladders of *b*- and *y*-ion series were computed. Calculated ion distributions were compared against experimental spectra, and the ion ladder leading to the best match was used to make spectral assignments.

2. Methods

2.1. pMBT-mediated cleavage of ΔAlaAz-containing peptides

Reagent stocks. Potassium phosphate (**KPi**) buffers were prepared by dissolving an appropriate amount of monobasic potassium phosphate in water (100 mM) and adjusting the pH with hydrochloric acid or aqueous potassium hydroxide as needed. Sodium acetate (pH 5.2) buffer was prepared by diluting a 3 M commercial stock to 100 mM with water. Buffers were stored at room temperature. pMBT stocks in dimethylformamide (**DMF**) were to prepared fresh every time. Peptides were dissolved in dimethylsulfoxide (**DMSO**) to 20–30 mM concentrations and stored at –20 or –80 °C. Prior to conducting a cleavage reaction, these DMSO stocks were diluted with water to 1.5 mM.

Standard procedure. In a 0.6 ml microcentrifuge tube were combined KPi buffer (26 µL), 1.5 mM ΔAlaAz-containing peptide in water/DMSO (2 µL), and 75 mM pMBT in DMF (2 µL). The contents were mixed by pipetting, and the tube was transferred to a 25 °C or 37 °C incubator as specified on a case-by-case basis. The tube was left capped and no stirring was performed. The final reaction conditions were thus 100 µM ΔAlaAz-containing peptide and 5 mM pMBT in KPi buffer (usually, pH 3.6 or 7.4) containing 6% (v/v) DMF and ca. 0.5% DMSO (v/v).

Reactions were allowed to proceed for 15 min unless otherwise specified. Then, an aliquot was diluted 20-fold into a water/acetonitrile mixture [30/70 (v/v) containing 0.1% TFA], and this sample was promptly analyzed by LC/MS as indicated in section 1.2.1.

Quenching. When quenching with N-methylmaleimide (**NMM**) was required (experiments in Fig. 3c, Fig. 4a, Fig. 4b, Fig. 6a, Fig. 6b, Fig. S2c, Fig. S4, S5 and Fig. S6), it was achieved by adding 1 volume of the quench solution to the reaction mixtures. The quench solution consisted of 30 mM NMM in KPi buffer/DMF mixture (1/1; buffer pH matched that of the reaction mixture).

Quenched reactions were incubated on ice for 10 min, after which LC/MS samples were prepared in a manner described above.

Glovebox reactions were conducted in an AtmosBag (Sigma Aldrich) that was subjected to four evacuation-backfill purge cycles with 99.999% pure nitrogen. All solvents and reagent stocks were degassed prior to bringing them into the glovebox. Otherwise, the reactions were performed as described in the standard procedure and were quenched with NMM after 15 min, except the tubes were left inside the glovebox at room temperature.

Reactions in the presence of additives. Solutions of dimedone in water (150 mM), sodium ascorbate in water (300 mM; pH adjusted to ca. 3.6), sodium cyanide in water (300 mM; pH adjusted to ca. 5.5), ethylenediaminetetraacetic acid in water (300 mM; pH adjusted to ca. 5.5), bis(para-methoxybenzene) disulfide in DMF (75 mM), and TEMPO in DMF (300 mM) were prepared freshly before use. In a 0.6 ml microcentrifuge tube were combined KPi buffer (24 μL; pH 3.6), 1.5 mM ΔAlaAz-containing peptide in water/DMSO (2 μL), 75 mM pMBT in DMF (2 μL), and the solution of an additive from above (2 μL). Reactions were then carried out as described above and quenched with NMM prior to analysis by LC/MS.

2.2. Thia-Michael conjugation of dhAA-containing peptides to cysteamine

Reactions were performed similarly to the procedure from section 2.1. In a 0.6 ml microcentrifuge tube were combined KPi buffer (26 μL; pH 7.4), 1.5 mM dhAA-containing peptide in water/DMSO (2 μL), and 75 mM cysteamine in water (2 μL; pH adjusted to neutral prior to use). The contents were mixed by pipetting, and the tube was transferred to a 37 °C incubator. Reactions were allowed to proceed for 4 h, and then, an aliquot was diluted 20-fold into a water/acetonitrile mixture [30/70 (v/v) containing 0.1% TFA]. This sample was analyzed by LC/MS as indicated in section 1.2.1.

2.3. Cell lysate recovery of Ac-HA peptide

In a low-binding 0.6 mL tube on ice were combined HeLa cell lysate (9.5 μL; Abcam #ab150035; 2 mg/mL total protein content) and 400 μM pep.04 in water (0.5 μL; final concentration: 20 μM). The contents4 were mixed well by pipetting and incubated at 4 °C for 30 min.

In the meantime, 50 μL of freshly resuspended M280 streptavidin Dynabeads (ThermoFisher #11205D) were washed with ice-cold HBS buffer (50 mM HEPES, 150 mM NaCl, pH 7.5) three times (3 × 100 μL). The supernatant was removed, and the lysate-pep.04 mixture was added to the beads. The mixture was homogenized by pipetting and incubated at 4 °C while gently rotating for 10 min, discarding the supernatant at the end.

The beads were washed five times with ice-cold HBS buffer containing 1 M urea (5 × 200 μL), and the supernatant was thoroughly removed after the last wash. The beads were then resuspended in KPi buffer [14 μL; pH 7.4; contained 0.5% (v/v) DMSO], and pMBT in DMF (1 μL of 75 mM stock; final concentration: 5 mM) was added. The contents were mixed well by pipetting and incubated for 15 min in a 37 °C thermostat. The supernatant was collected, diluted 7-fold into

a water/acetonitrile mixture [30/70 (v/v) containing 0.1% TFA], and analyzed by LC/MS as described in section 1.2.1.

2.4. Small molecule reactions

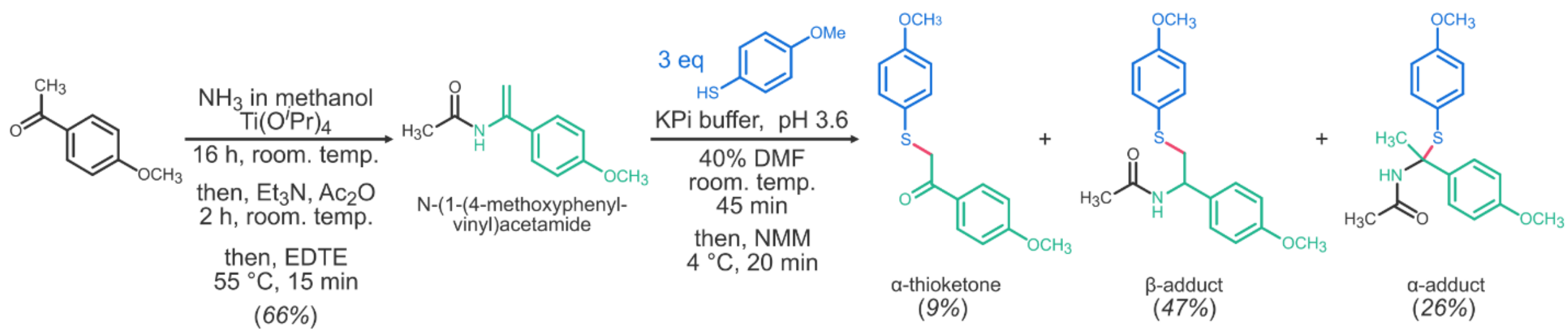

N-(1-(4-methoxyphenyl)vinyl)acetamide. The known compound[1] was synthesized by modifying a published procedure.[1] In a flame-dried round bottom flask equipped with a magnetic stir bar was dissolved 4-methoxyacetophenone (601 mg; 4 mmol) in dry toluene (2.4 mL). The flask was capped, flushed with nitrogen three times, and left under slight positive pressure of nitrogen. The flask was cooled down in an ice bath, and solution of ammonia in methanol (7 M; 6 mmol; 857 µL) was added via a syringe in one portion with constant stirring. Then, titanium tetraisopropoxide (8 mmol; 2.37 mL) was added dropwise via a syringe. After 10 min, the ice bath was removed, and the flask was left to warm to room temperature while continuing stirring for 16 h. Then, the flask was placed in an ice bath again; triethylamine (16 mmol; 2.23 mL) and acetic anhydride (8 mmol; 755 µL) were both added via a syringe in one portion in sequence. The ice bath was removed, and the flask was left to warm to room temperature while continuing stirring for 1 h. Finally, to the flask was added N,N,N′,N′-tetrakis(2-hydroxyethyl)ethylenediamine (EDTE; 8.4 mmol; 1.80 mL), and the mixture was heated in an oil bath (bath temperature: 55 °C) for 15 min while maintaining constant stirring. The reaction mixture was poured into a separatory funnel containing ethyl acetate (20 mL), water (12 mL), and saturated aqueous ammonium hydroxide (4 mL). The layers were separated, and the aqueous layer was extracted once more with ethyl acetate (20 mL). Combined organic layers were dried over anhydrous sodium sulfate, evaporated under reduced pressure, and the residue was purified by silica gel column chromatography using ethyl acetate / *n*-hexane as the solvent system. The product was isolated as a colorless solid (503 mg, 2.63 mmol, 66%).

^{1}H NMR (400 MHz, DMSO-d_6) δ 9.21 (s, 1H), 7.44 – 7.30 (m, 2H), 6.99 – 6.84 (m, 2H), 5.52 (s, 1H), 4.90 (s, 1H), 3.76 (s, 3H), 2.00 (s, 3H).

^{13}C NMR (101 MHz, DMSO-d_6) δ 169.0, 159.4, 141.0, 130.4, 127.5, 113.6, 100.5, 55.2, 40.1, 39.9, 39.7, 39.5, 39.3, 39.1, 38.9, 23.8.

Reaction between N-(1-(4-methoxyphenyl)vinyl)acetamide and pMBT. In a 15 mL Falcon tube was dissolved N-(1-(4-methoxyphenyl)vinyl)acetamide (96 mg; 0.5 mmol) in a mixture of KPi buffer (3.5 mL; pH 3.6) and DMF (2.5 mL). pMBT was then added (1.5 mmol; 184 µL); the tube was capped, shaken vigorously to mix the contents, and agitated on a rotary mixer at room

temperature for 45 min, upon which TLC indicated full consumption of the starting material. N-methylmaleimide (1.5 mmol; 166 mg) was dissolved in DMF, and added to the reaction mixture. The tube was transferred on ice, and left at that temperature for 20 min. The mixture was poured into a separatory funnel containing ethyl acetate (15 mL) and water (10 mL). The organic layer was washed twice with aqueous solution of lithium chloride (5%, w/v; 2 × 5 mL), and dried over anhydrous sodium sulfate. The solvent was evaporated under reduced pressure, and the residue was purified by silica gel column chromatography using ethyl acetate / *n*-hexane as the solvent system to separate the products. Three pure compounds were isolated as colorless oils: the **α-thioketone** [1-(4-methoxyphenyl)-2-((4-methoxyphenyl)thio)ethan-1-one, 13 mg, 0.045 mmol, 9%], the **β-adduct** [N-(1-(4-methoxyphenyl)-2-((4-methoxyphenyl)thio)ethyl)acetamide, 78 mg, 0.235 mmol, 47%], and the **α-adduct**, N-(1-(4-methoxyphenyl)-1-((4-methoxyphenyl)thio)ethyl)-acetamide, 44 mg, 0.132 mmol, 26%].

α-thioketone, 1-(4-methoxyphenyl)-2-((4-methoxyphenyl)thio)ethan-1-one. Spectral data matches refs. 2 and 3.

^{1}H NMR (400 MHz, $CDCl_3$) δ 7.97 – 7.83 (m, 2H), 7.42 – 7.32 (m, 2H), 7.00 – 6.88 (m, 2H), 6.86 – 6.77 (m, 2H), 4.09 (s, 2H), 3.87 (s, 3H), 3.78 (s, 3H).

^{13}C NMR (101 MHz, $CDCl_3$) δ 193.2, 163.8, 159.8, 134.7, 131.2, 128.6, 125.0, 114.8, 113.9, 55.6, 55.4, 42.7.

TOF-HRMS (*m/z*): calcd for $C_{16}H_{16}NaO_3S^+$ $[M+Na]^+$: 311.0712, found: 311.0705.

β-adduct, N-(1-(4-methoxyphenyl)-2-((4-methoxyphenyl)thio)ethyl)acetamide.

^{1}H NMR (400 MHz, $CDCl_3$) δ 7.39 – 7.27 (m, 2H), 7.21 – 7.09 (m, 2H), 6.89 – 6.76 (m, 4H), 6.13 (d, *J* = 7.8 Hz, 1H), 5.04 (q, *J* = 7.3 Hz, 1H), 3.77 (s, 3H), 3.77 (s, 3H), 3.32 – 3.09 (m, 2H), 1.94 (s, 3H).

^{13}C NMR (101 MHz, $CDCl_3$) δ 169.6, 159.2, 159.2, 133.6, 132.8, 127.9, 125.7, 114.8, 114.2, 55.4, 55.4, 52.5, 41.8, 23.4.

TOF-HRMS (*m/z*): calcd for $C_{18}H_{21}NNaO_3S^+$ $[M+Na]^+$: 354.1134, found: 354.1129.

α-adduct, N-(1-(4-methoxyphenyl)-1-((4-methoxyphenyl)thio)ethyl)acetamide.

^{1}H NMR (400 MHz, $CDCl_3$) δ 7.32 – 7.25 (m, 2H), 7.25 – 7.18 (m, 2H), 6.87 – 6.76 (m, 4H), 6.16 (s, 1H), 3.79 (s, 3H), 3.78 (s, 3H), 2.03 (s, 3H), 1.95 (s, 3H).

^{13}C NMR (101 MHz, $CDCl_3$) δ 168.8, 161.0, 158.8, 138.6, 135.0, 127.1, 122.2, 114.5, 113.5, 68.6, 55.4, 55.3, 27.6, 24.3.

TOF-HRMS (*m/z*): calcd for $C_{18}H_{21}NNaO_3S^+$ $[M+Na]^+$: 354.1134, found: 354.1127.

2.5. Peptide synthesis

The syntheses of peptides pep.01–03 were previously reported,[4,5] and are reproduced here for completeness.

Peptide synthesis was performed manually on NovaPEG Rink Amide resin (Novabiochem; ~0.4 mmol/g loading) or Fmoc-Rink Amide AM Resin (Aaptech; ~0.5 mmol/g) using the standard Fmoc/tBu chemistry. Side chain protection was as follows: Arg(Pbf), Asn(Trt), Asp(OtBu), Gln(Trt), Glu(OtBu), His(Trt), Lys(Boc), Ser(tBu), Thr(tBu), Trp(Boc), and Tyr(tBu). Dehydroamino acids were accessed following a late stage oxidative elimination conducted on *Se*-substituted Sec-containing precursor peptides. The synthesis of the Fmoc-protected *Se*-containing amino acids [Fmoc–Sec(*p*-MeBzl)–OH, Fmoc-Sec(Ph)-Oxz-OH, and Fmoc-Sec(Ph)-Thz-OH] used as precursors to ΔAla, ΔAlaOxz, and ΔAlaThz, respectively, was as previously described.[4,6–8]

After swelling the resin for 5 min with anhydrous dichloromethane (DCM; 2 mL), iterative cycles of amino acid coupling and Fmoc deprotection were initiated. Coupling conditions were as follows: Fmoc-amino acid (10 eq.), 1-[bis(dimethylamino)methylene]-1*H*-1,2,3triazolo[4,5-b]pyridinium 3-oxide hexafluorophosphate (HATU; 9.5 eq.) and *N,N*-diisopropylethylamine (DIPEA; 20 eq.) in DMF (0.4 M activated amino acid ester) for 15 min at room temperature. The coupling of custom *Se*-containing amino acids was conducted using 2.5 eq. amino acid scaling the amount of HATU and DIPEA accordingly and letting the coupling proceed overnight. Fmoc deprotection was performed using piperidine in DMF (1.5 mL; 20% [v/v]; 3 min × 2).

After the peptidyl chain assembly, the resin was washed with DMF (1.5 mL × 3) and DCM (3 mL × 3), and briefly dried under reduced pressure. A freshly prepared deprotection reagent consisting of trifluoroacetic acid (94% [v/v]), ethanedithiol (2.5%), water (2.5%) and triisopropylsilane (1%) was added to the resin (3 mL) at room temperature, and the mixture was left to stand for 2 h. To the resulting suspension was added ice-cold diethyl ether (45 mL) to precipitate the peptide. After centrifugation (5000× g; 5 min), the liquid was decanted, and to the residue was added ice-cold diethyl ether (5 mL). The liquid was decanted after centrifugation as above, and this procedure was repeated twice more. The residue was dissolved in a water/acetonitrile mixture (30/70 (v/v) containing 1% (v/v) trifluoroacetic acid; 10–15 mL), filtered through a 0.45 μm syringe filter to remove the resin, and lyophilized to dryness.

Oxidative elimination and purification. In a 50 ml Falcon tube, crude *Se*-containing peptide was dissolved in dimethyl sulfoxide to the final concentration of ca. 20 mM, and a solution of hydrogen peroxide in phosphate buffer (100 mM hydrogen peroxide in 100 mM sodium phosphate buffer, pH 8.0; 4.33 volumes w.r.t. the dimethyl sulfoxide volume) was added. The mixture was stirred on an orbital shaker for 120 min at room temperature. The solution was filtered through a 0.22 μM syringe filter and purified by RP-HPLC. Fractions containing pure product were pooled and lyophilized to dryness. Analytical data for the purified peptides is shown in section 5.2.

For pep.04, the concentration of hydrogen peroxide was decreased to 80 mM to minimize the undesired oxidation of biotin.

3. Supplementary Figures

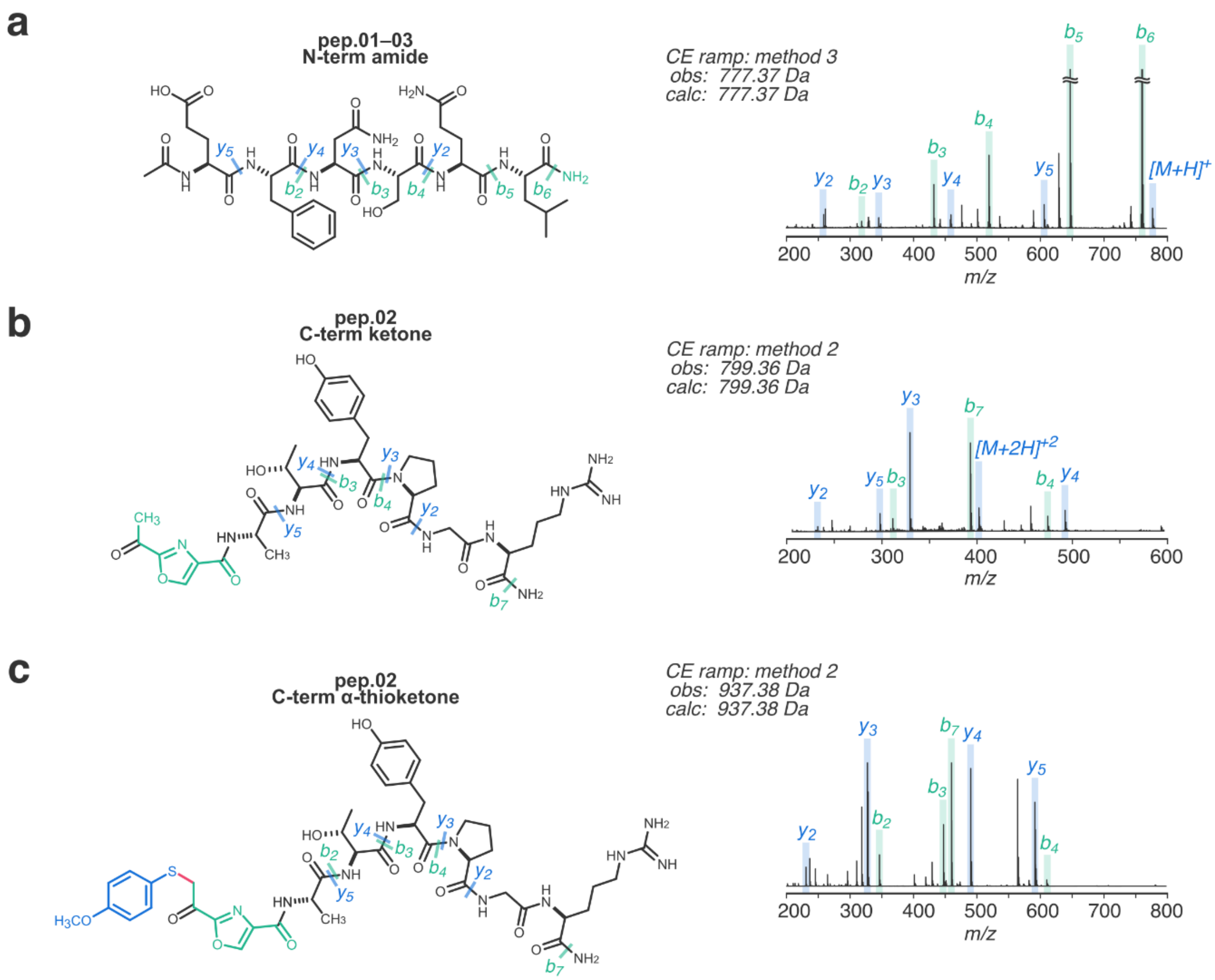

Figure S1. MS/MS characterization of the cleavage products shown in Fig. 2c. Displayed are CID fragmentation spectra with *b*- and *y*-ion fragmentations annotated; neutral molecule losses are omitted for clarity. **b**) The assigned chemical structures of the associated products are shown on the left with the observed fragmentation sites indicated. **a**) The spectrum for pep.01–03 N-term amide. **b**) The spectrum for pep.02 C-term ketone. **c**) The spectrum for pep.02 C-term α-thioketone.

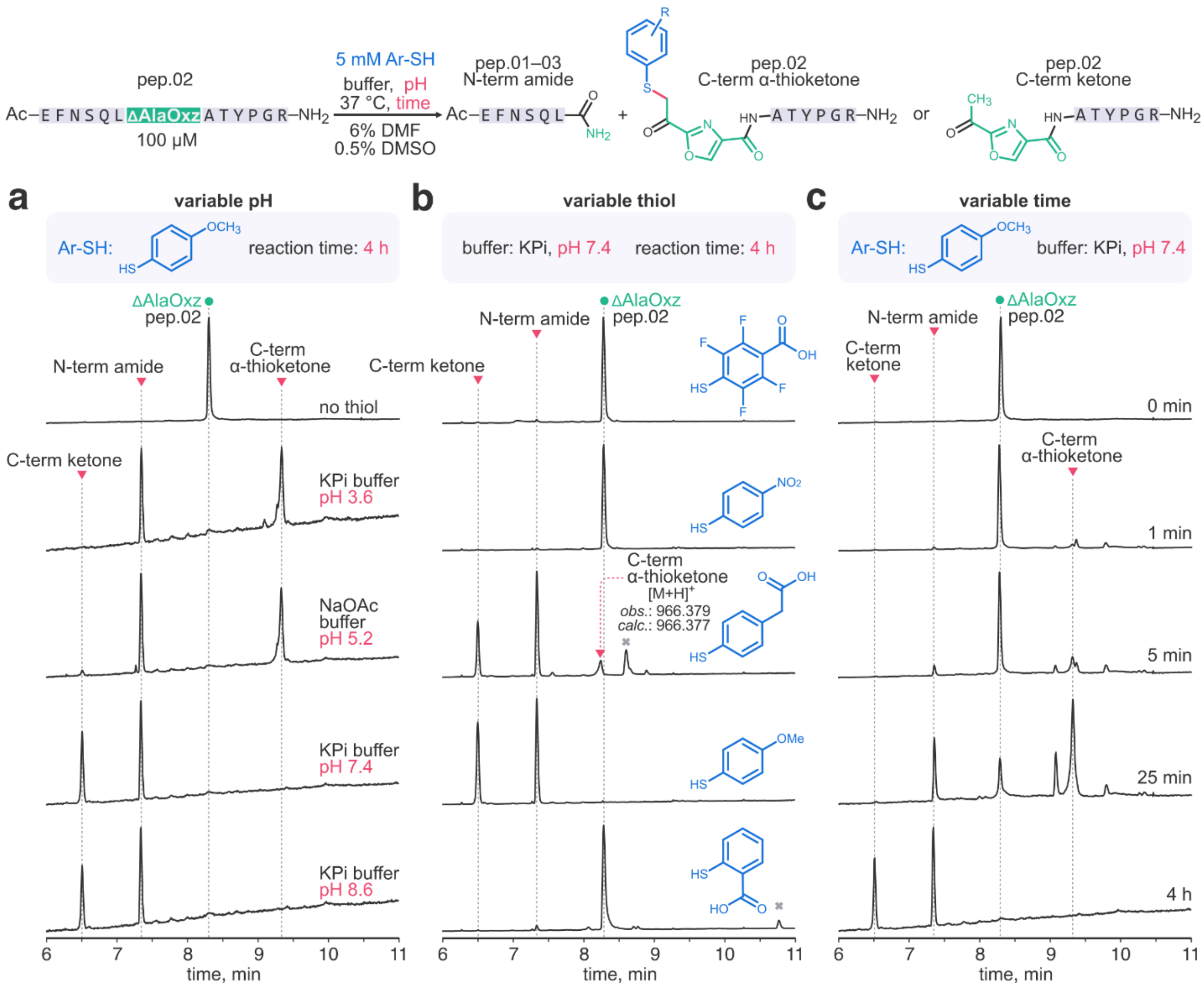

Figure S2. Additional reaction mechanism investigations. Peptide pep.02 was incubated with aryl thiols as indicated in the scheme, and the reaction outcomes were analyzed by LC/MS. See section 2.1 for reaction protocol details. In each case, displayed are the annotated TIC LC/MS chromatograms showing reaction outcomes for the pH (panel **a**), aryl thiol (**b**) and time course (**c**) studies. Peaks labelled with a grey cross (×) were not derived from the peptide analytes. Analogous data for pep.03 is shown in Fig. 3.

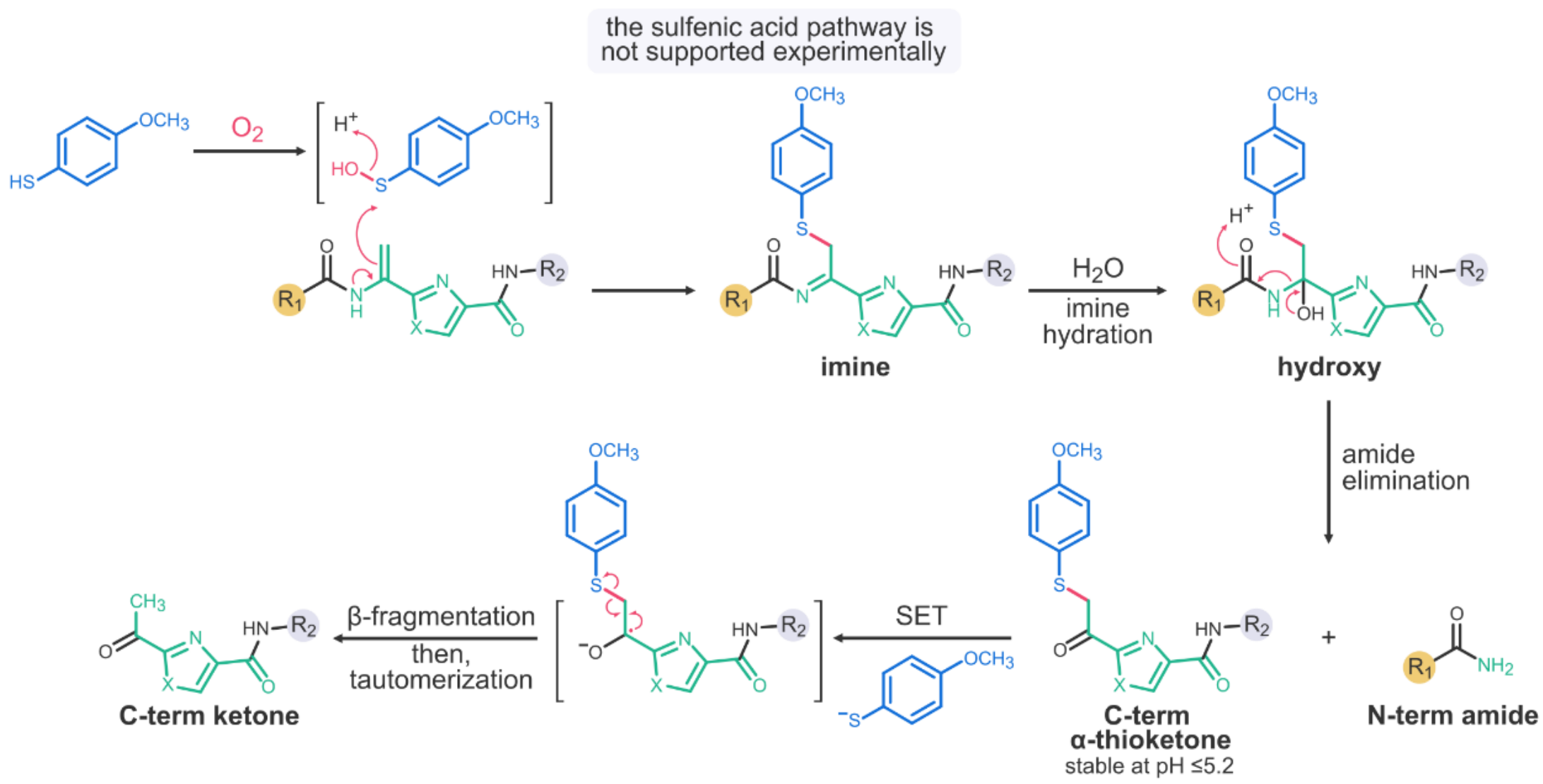

Figure S3. The sulfenic acid pathway to the observed products. This pathway is not supported by experimental data. A plausible mechanism is shown in Fig. 5; see the main text for additional discussion.

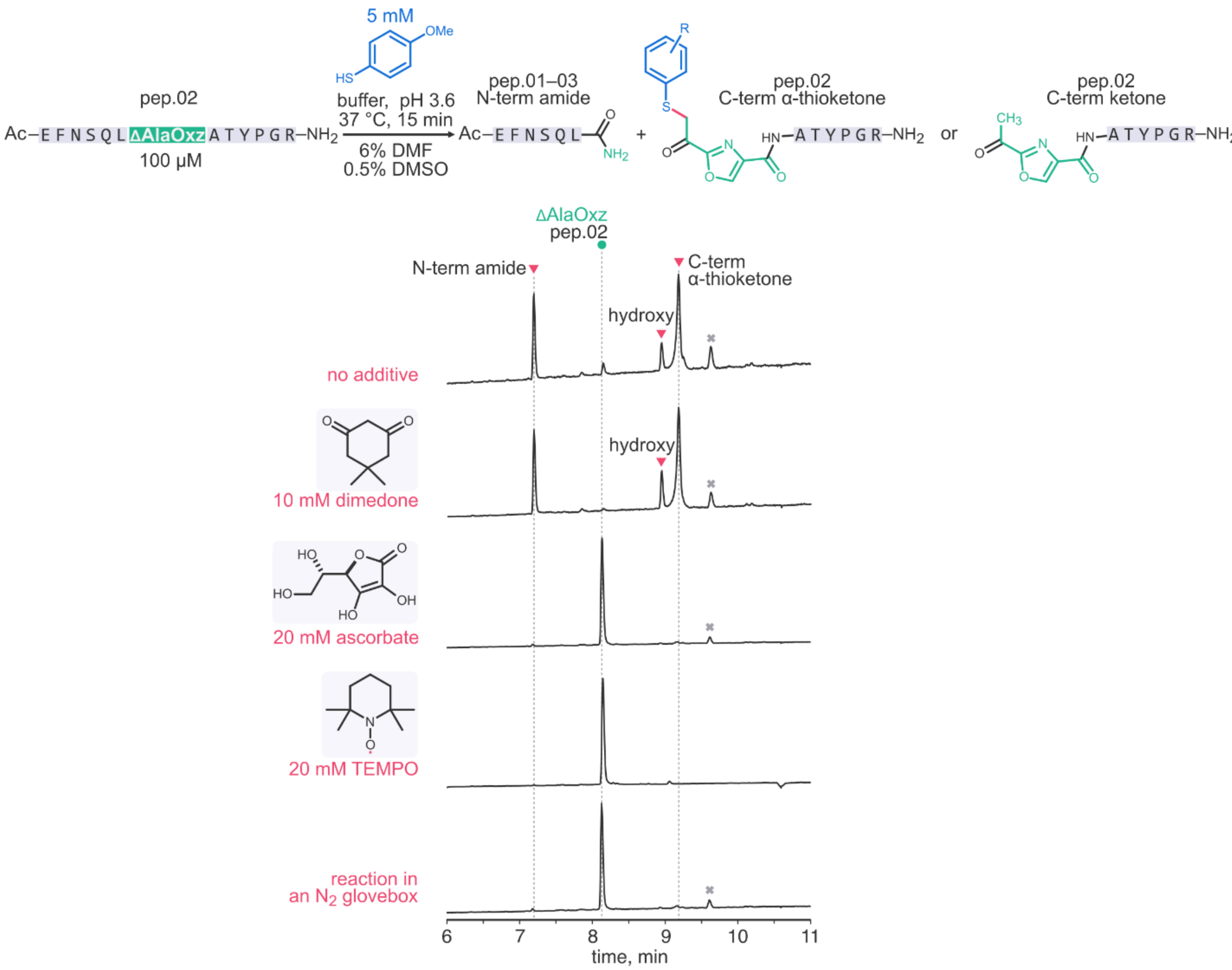

Figure S4. Additional reaction mechanism investigations. Peptide pep.02 was incubated with pMBT in the presence of various additives or in a glovebox as indicated in the scheme, and the reaction outcomes were analyzed by LC/MS. In each case, displayed are the annotated TIC LC/MS chromatograms showing reaction outcomes. See section 2.1 for reaction protocol details. Peaks labelled with a grey cross (×) were not derived from the peptide analytes. Analogous data for pep.03 is shown in Fig. 4a.

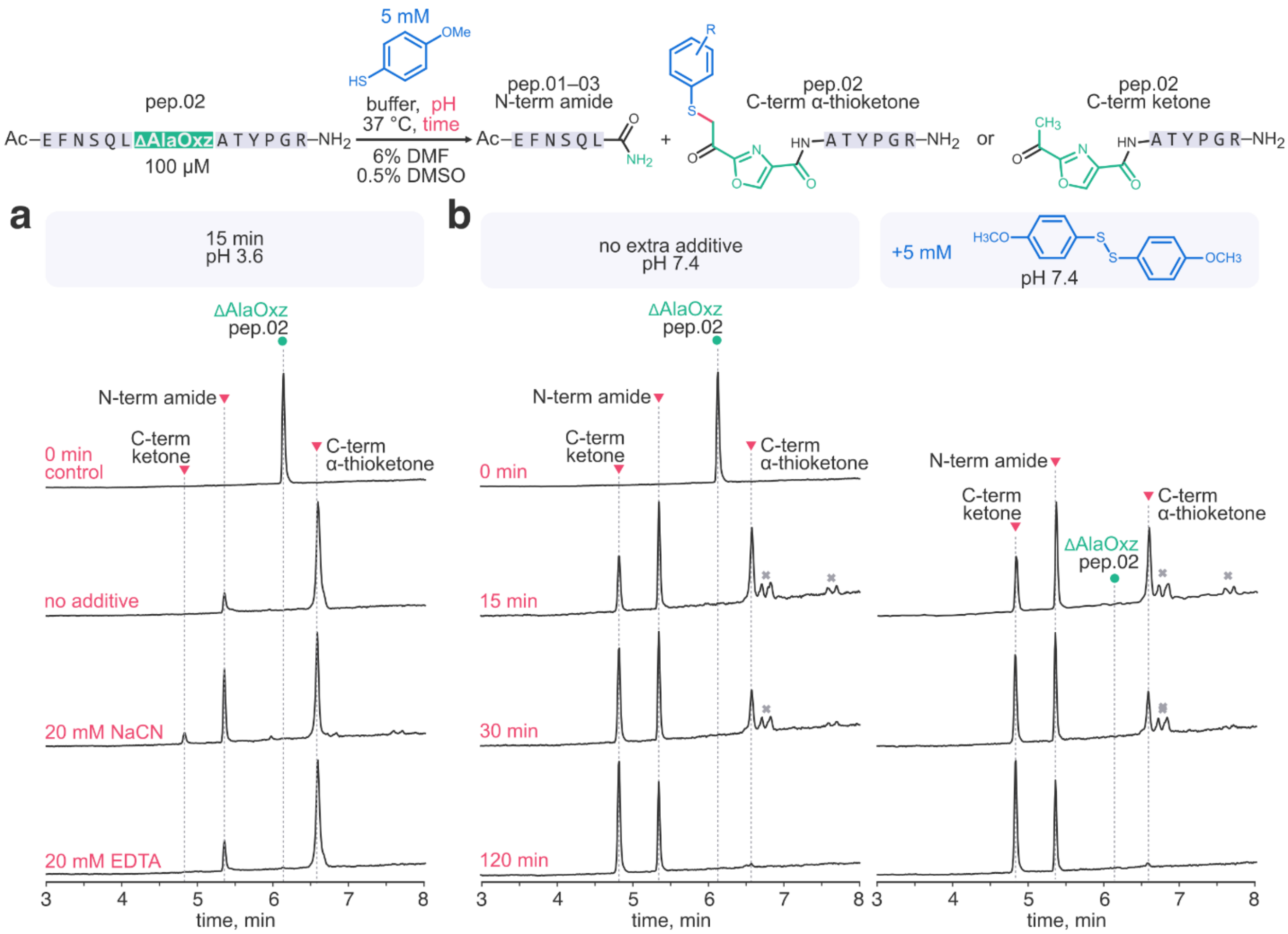

Figure S5. Additional reaction mechanism investigations. Peptide pep.02 was incubated with pMBT in the presence of various additives as indicated in the scheme, and the reaction outcomes were analyzed by LC/MS. In each case, displayed are the annotated TIC LC/MS chromatograms showing reaction outcomes. See section 2.1 for reaction protocol details. Peaks labelled with a grey cross (×) indicate quencher-derived byproducts. **a**) Reactions in the presence of metal scavenging species (sodium cyanide and EDTA) suggest catalysis by transition metals is unlikely to play a critical role in the cleavage. **b**) Reactions with externally added pMBT disulfide. The addition of this disulfide did not change the rate of the C-term α-thioketone reduction, suggesting that the disulfide is not the primary reducing agent in the process.

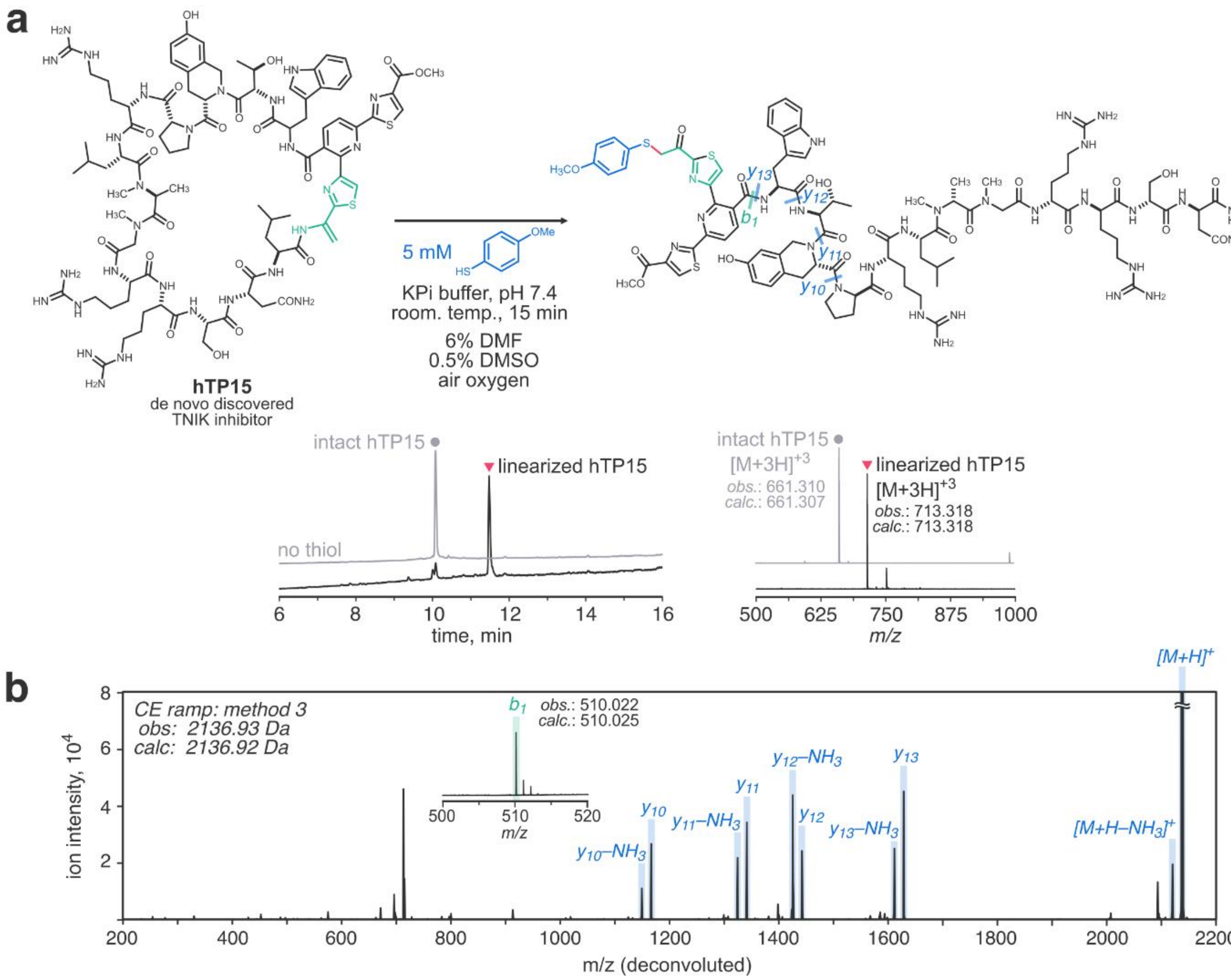

Figure S6. Linearization of thiopeptide hTP15 with pMBT. **a**) The reaction was performed as indicated in the scheme. See section 2.1 for reaction protocol details. Displayed are the annotated TIC LC/MS chromatograms showing the reaction outcomes in the presence (black) or absence (grey) of pMBT. The associated ESI-MS spectra for the biggest chromatogram peak are shown on the right. Analogous data for TP15 is shown in Fig. 6a. **b**) MS/MS characterization of the linearized hTP15 from panel a). Shown is the charge-deconvoluted CID fragmentation spectrum with *b*- and *y*-ion fragmentation annotations; the low intensity ion corresponding to the key b_1 ion is shown as an inset. The assigned chemical structure is supported by the observed MS/MS fragmentation pattern.

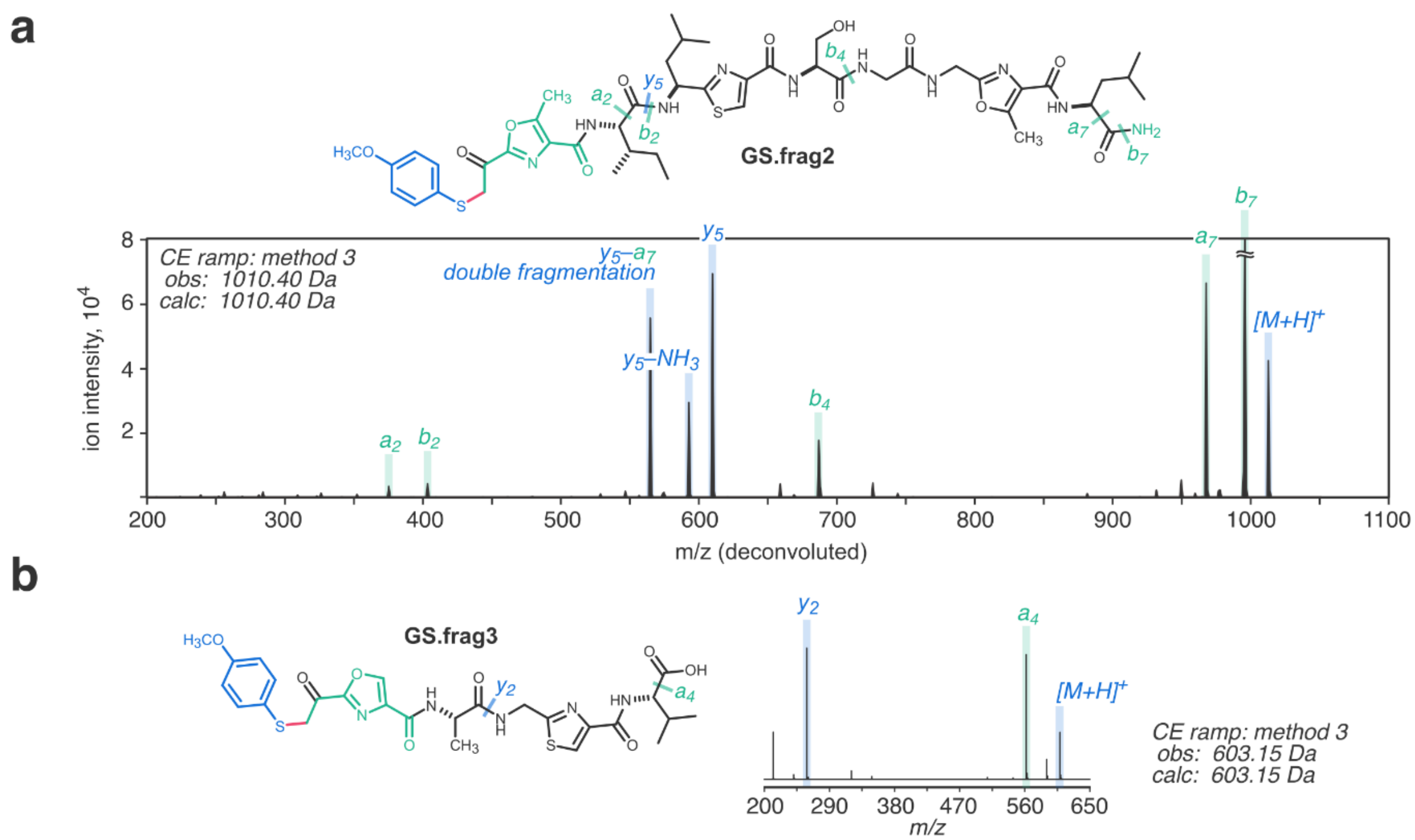

Figure S7. Fragmentation of goadsporin with pMBT. See Fig. 6b for additional data. **a**) MS/MS characterization of GS.frag2. Shown is the charge-deconvoluted CID fragmentation spectra with *b*- and *y*-ion fragmentation annotations. **b**) MS/MS characterization of GS.frag2. Shown is the CID fragmentation spectra with major fragmentations annotated. In both cases, the assigned chemical structures are supported by the observed MS/MS fragmentation patterns.

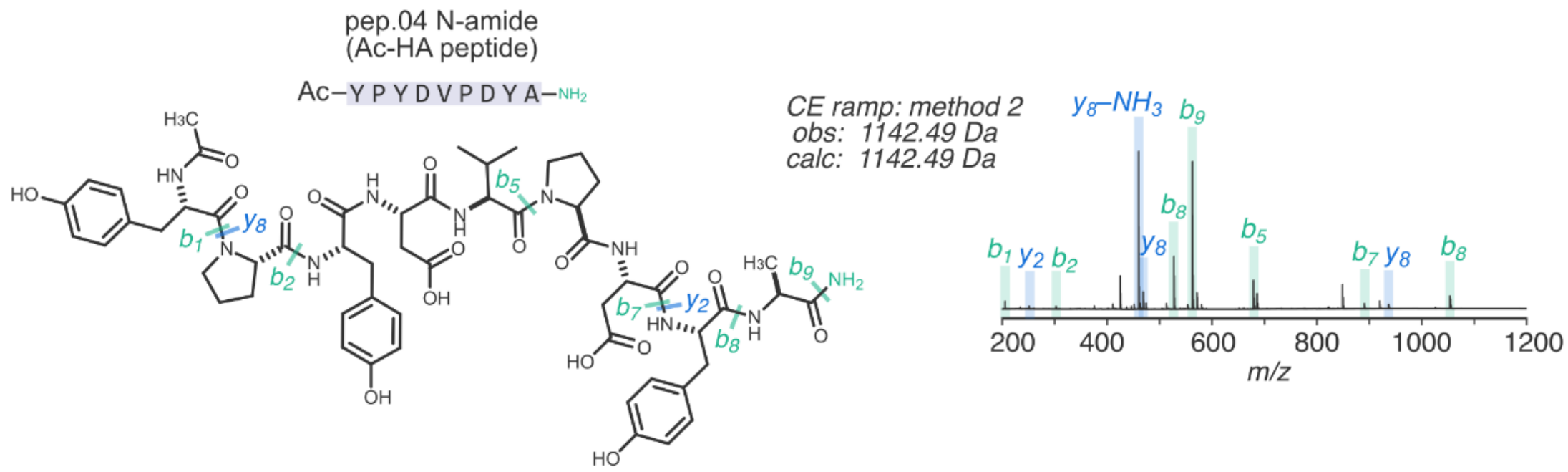

Figure S8. MS/MS characterization of pep.04 N-term amide (Ac-HA peptide) recovered in the cell lysate experiment. See Fig. 6c and section 2.3 for further details. Shown is the CID fragmentation spectra with *b*- and *y*-ion fragmentation annotations. The assigned chemical structure is supported by the observed MS/MS fragmentation patterns.

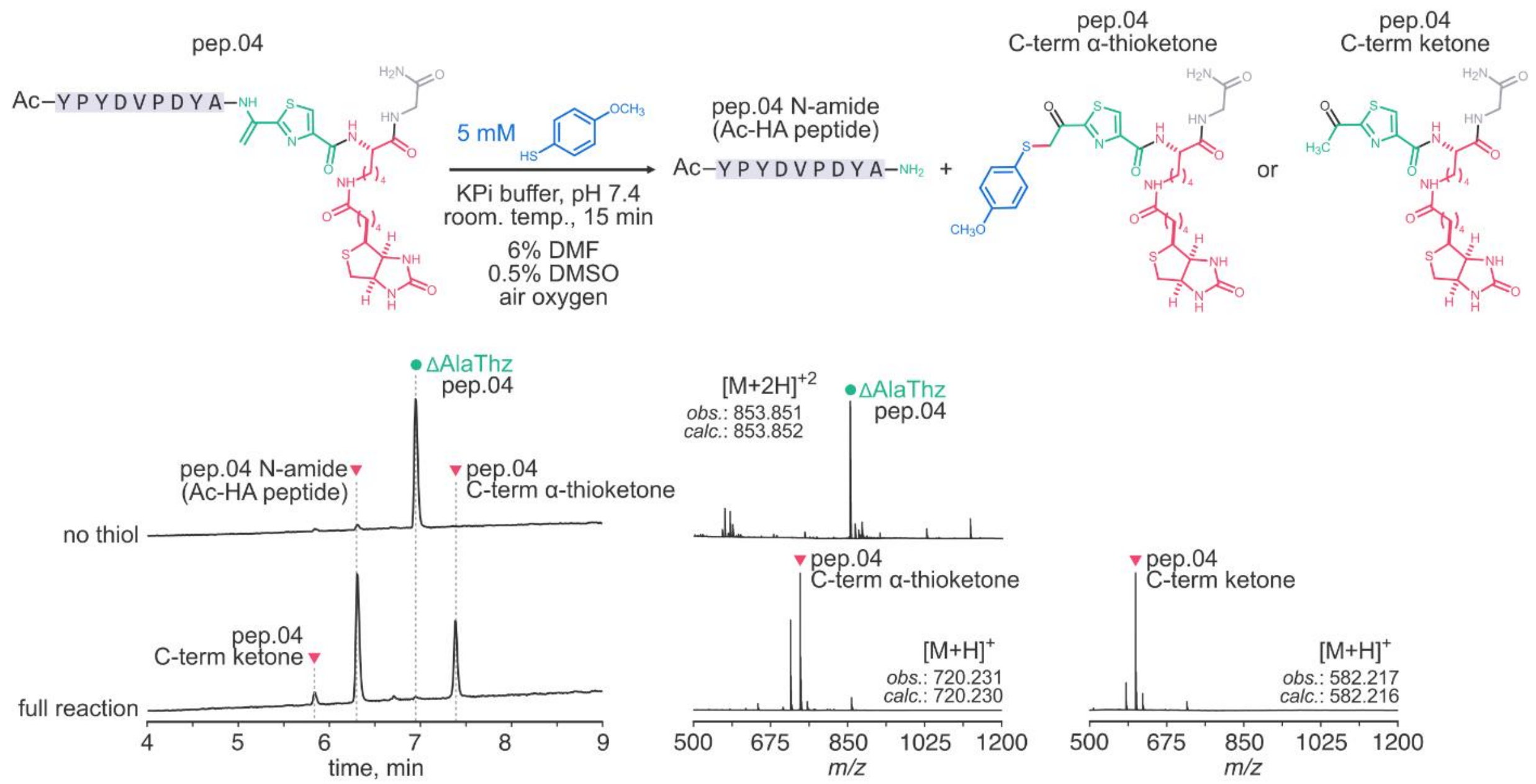

Figure S9. Cleavage of pep.04 in solution. Peptide pep.04 was incubated with pMBT as indicated in the scheme and reaction outcomes were analyzed by LC/MS. See S.I. 2.1 for reaction protocol details. Displayed are the annotated TIC LC/MS chromatograms showing the reaction outcomes in the presence (bottom) or absence (top) of pMBT. The associated ESI-MS spectra for the major reaction products are shown on the right.

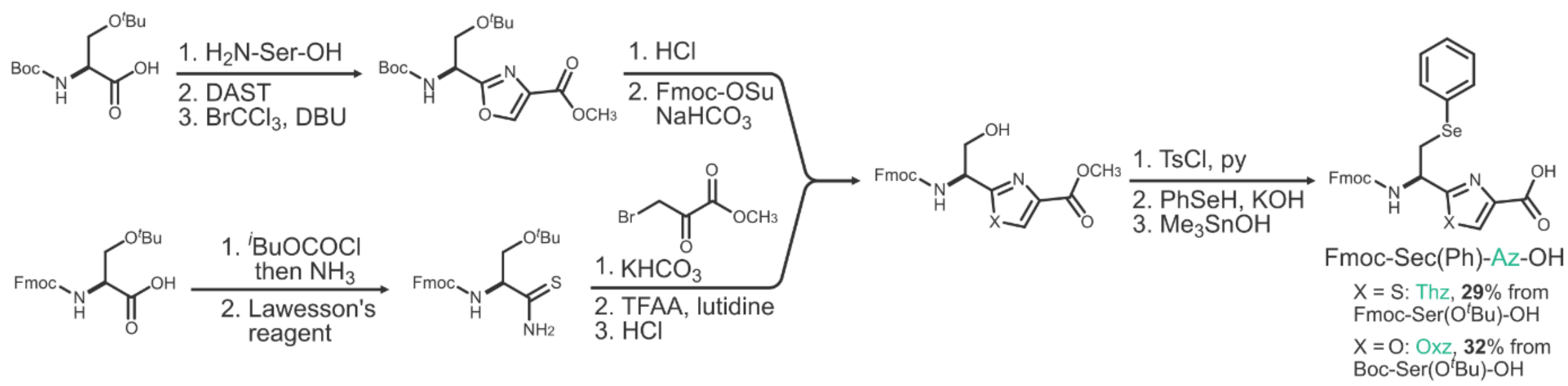

Figure S10. Synthesis of ΔAlaOxz and ΔAlaThz precursor amino acids for Fmoc SPPS. Complete experimental procedures are reported in refs. 4, 7, and 8.

4. References

(1) Reeves, J. T.; Tan, Z.; Han, Z. S.; Li, G.; Zhang, Y.; Xu, Y.; Reeves, D. C.; Gonnella, N. C.; Ma, S.; Lee, H.; Lu, B. Z.; Senanayake, C. H. Direct Titanium-Mediated Conversion of Ketones into Enamides with Ammonia and Acetic Anhydride. *Angew. Chem., Int. Ed* **2012**, *51*, 1400–1404.

(2) Dias, R. M. P.; Burtoloso, A. C. B. Catalyst-Free Insertion of Sulfoxonium Ylides into Aryl Thiols. A Direct Preparation of β-Keto Thioethers. *Org. Lett.* **2016**, *18*, 3034–3037.

(3) Pan, Y. Z.; Cheng, S. Y.; Li, Q. Y.; Tang, H. T.; Pan, Y. M.; Meng, X. J.; Mo, Z. Y. Electrocatalytic Synthesis of Gem -Bisarylthio Enamines and α-Phenylthio Ketones via a Radical Process under Mild Conditions. *Synlett* **2021**, *32*, 593–600.

(4) Vinogradov, A. A.; Pan, S. Y.; Suga, H. Ligand-Enabled Selective Coupling of MIDA Boronates to Dehydroalanine-Containing Peptides and Proteins. *J. Am. Chem. Soc.* **2025**, *147*, 7533−7544.

(5) Vinogradov, A. A.; Bashiri, G.; Suga, H. Illuminating Substrate Preferences of Promiscuous F420H2-Dependent Dehydroamino Acid Reductases with 4-Track mRNA Display. *J. Am. Chem. Soc.* **2024**, *146*, 31124−31136.

(6) Zhang, Y.; Vinogradov, A. A.; Chang, J. S.; Goto, Y.; Suga, H. Solid-Phase-Based Synthesis of Lactazole-Like Thiopeptides. *Org. Lett.* **2022**, *24*, 7894–7899.

(7) Chang, J. S.; Vinogradov, A. A.; Zhang, Y.; Goto, Y.; Suga, H. Deep Learning-Driven Library Design for the De Novo Discovery of Bioactive Thiopeptides. *ACS Cent. Sci.* **2023**, *9*, 2150–2160.

(8) Vinogradov, A. A.; Zhang, Y.; Hamada, K.; Kobayashi, S.; Ogata, K.; Sengoku, T.; Goto, Y.; Suga, H. A Compact Reprogrammed Genetic Code for De Novo Discovery of Proteolytically Stable Thiopeptides. *J. Am. Chem. Soc.* **2024**, *146*, 8058–8070.

5. Characterization data

5.1. NMR spectra

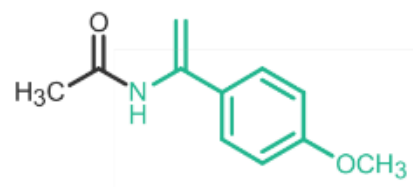

N-(1-(4-methoxyphenyl-vinyl)acetamide

^{1}H NMR of N-(1-(4-methoxyphenyl-vinyl)acetamide (400 MHz, DMSO-d_6)

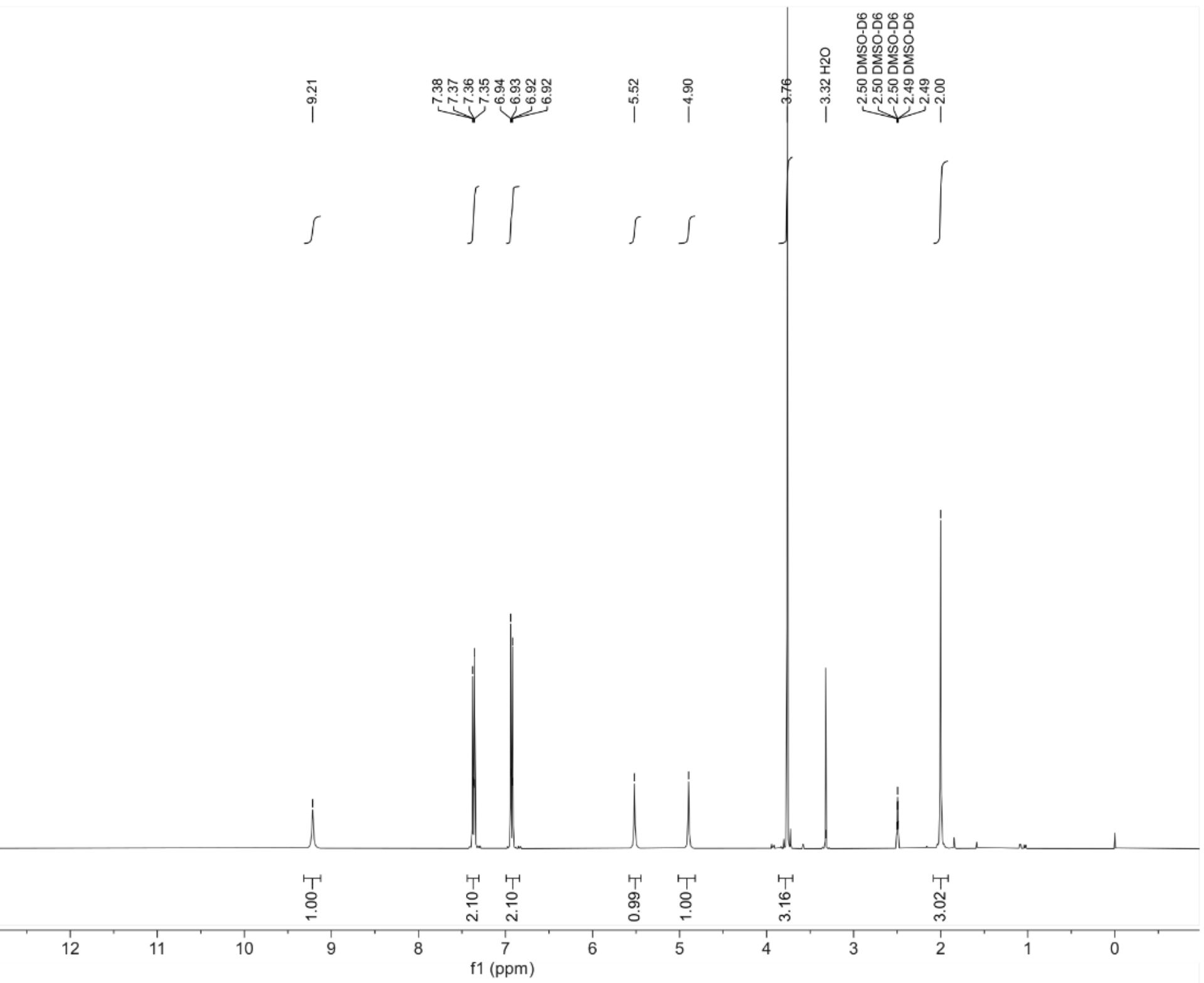

^{13}C NMR of N-(1-(4-methoxyphenyl-vinyl)acetamide (101 MHz, DMSO-d_6)

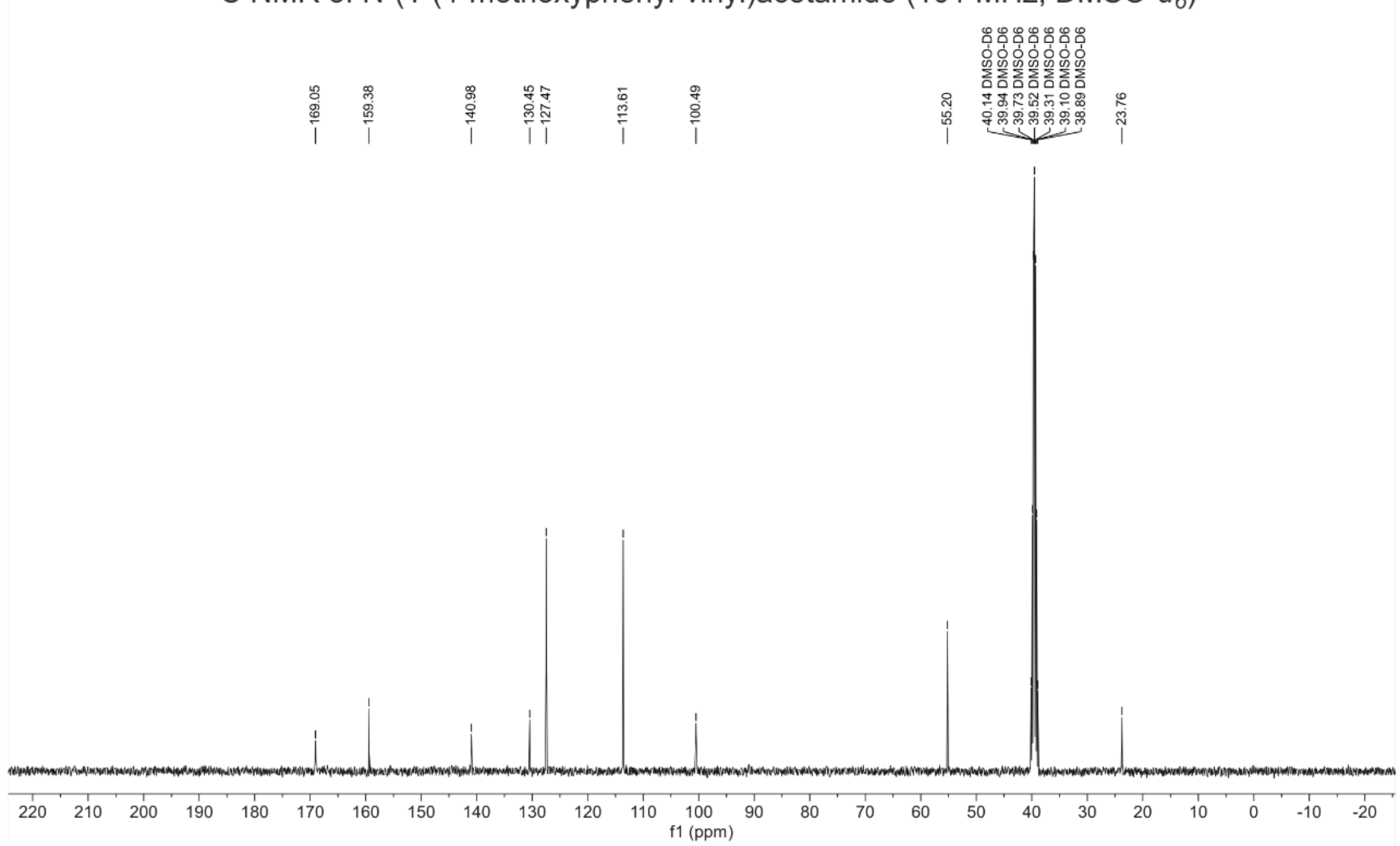

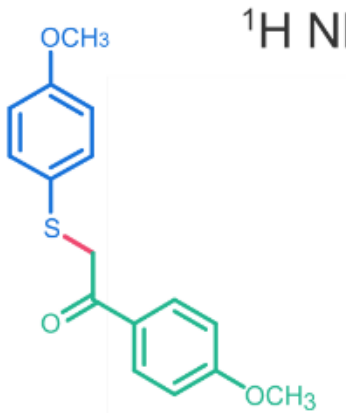

α-thioketone

^{1}H NMR of 1-(4-methoxyphenyl)-2-((4-methoxyphenyl)thio)ethan-1-one (400 MHz, $CDCl_3$)

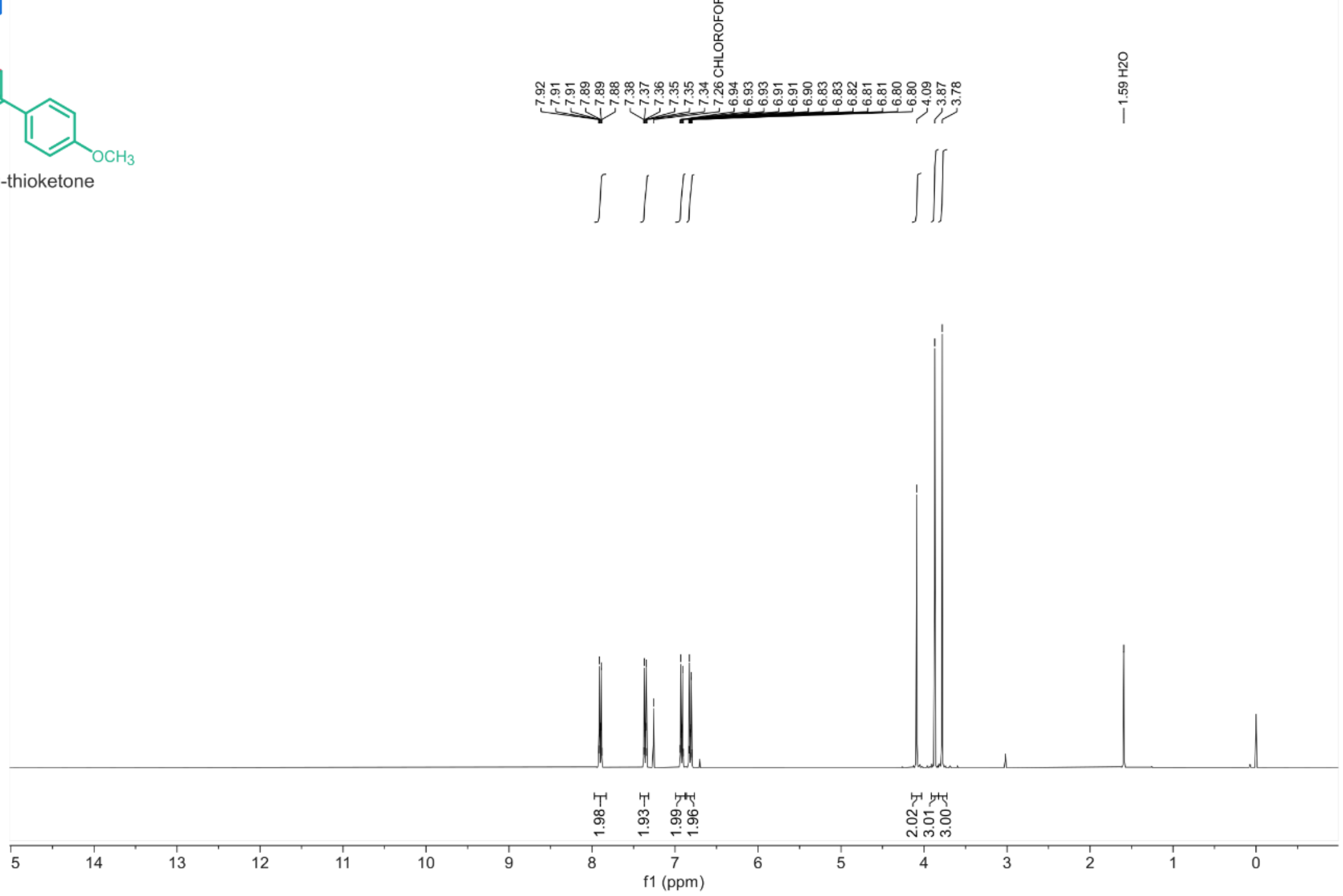

^{13}C NMR of 1-(4-methoxyphenyl)-2-((4-methoxyphenyl)thio)ethan-1-one (101 MHz, $CDCl_3$)

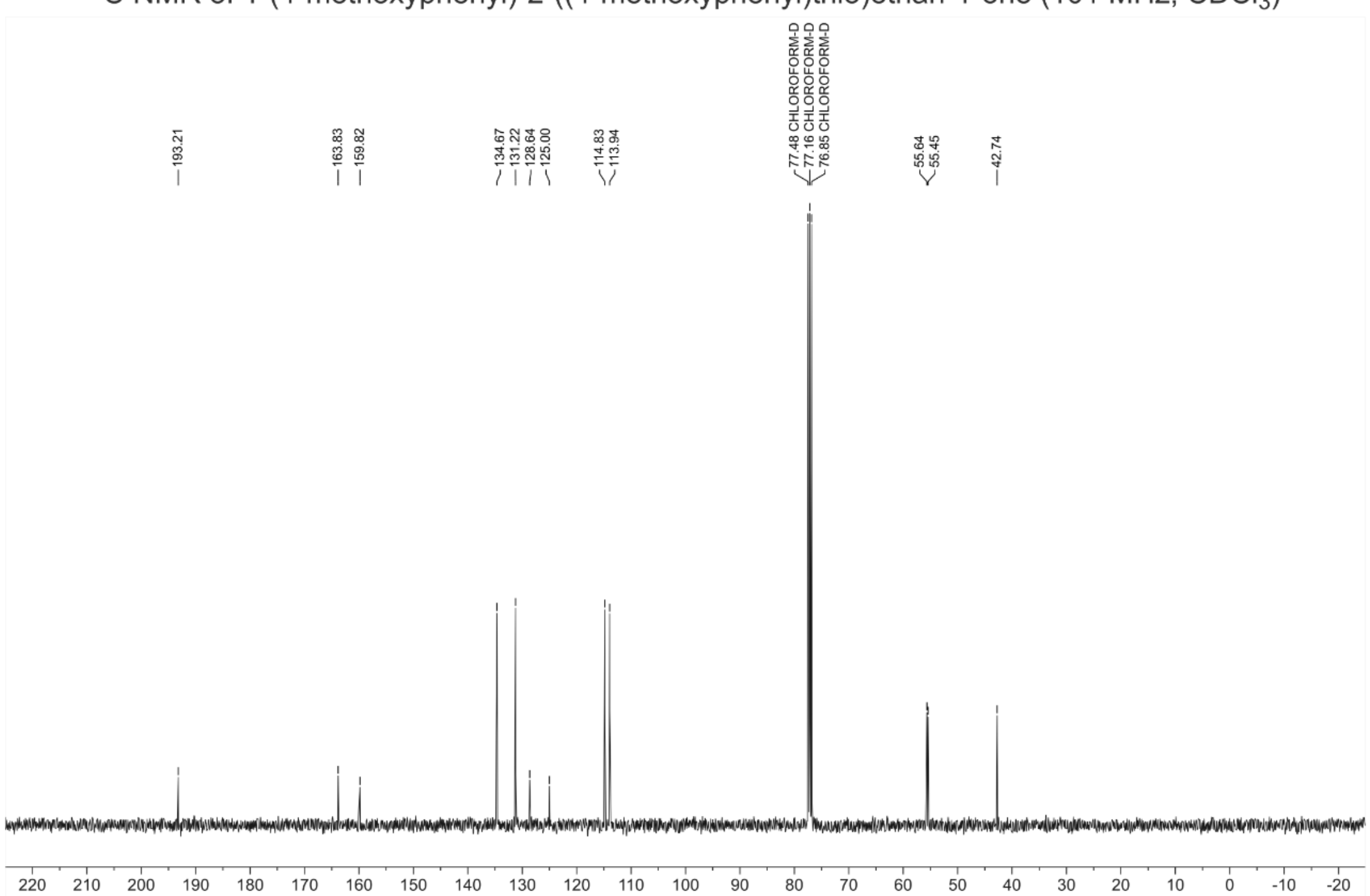

^{1}H NMR of N-(1-(4-methoxyphenyl)-2-((4-methoxyphenyl)thio)ethyl)acetamide (400 MHz, $CDCl_3$)

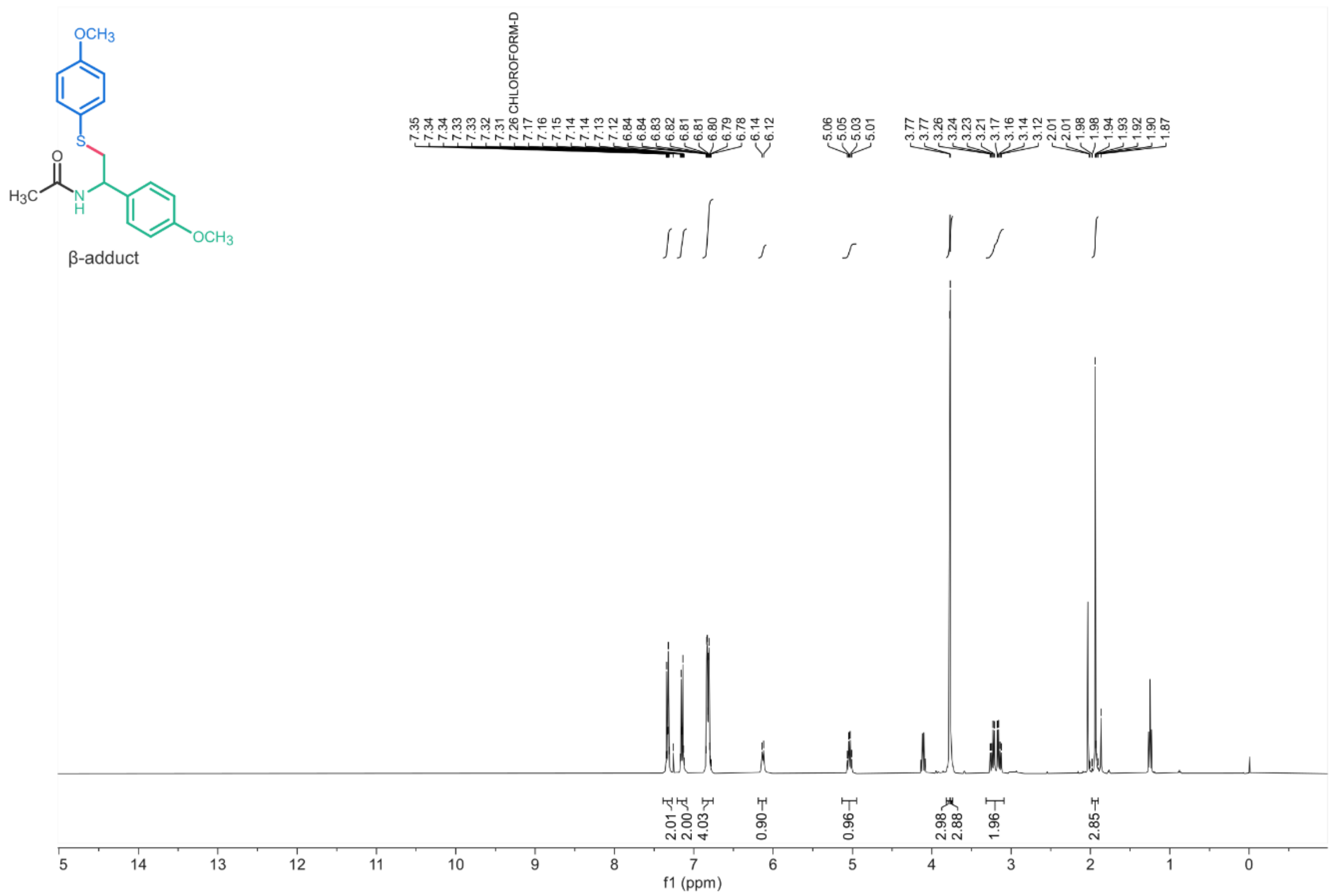

^{13}C NMR of N-(1-(4-methoxyphenyl)-2-((4-methoxyphenyl)thio)ethyl)acetamide (101 MHz, $CDCl_3$)

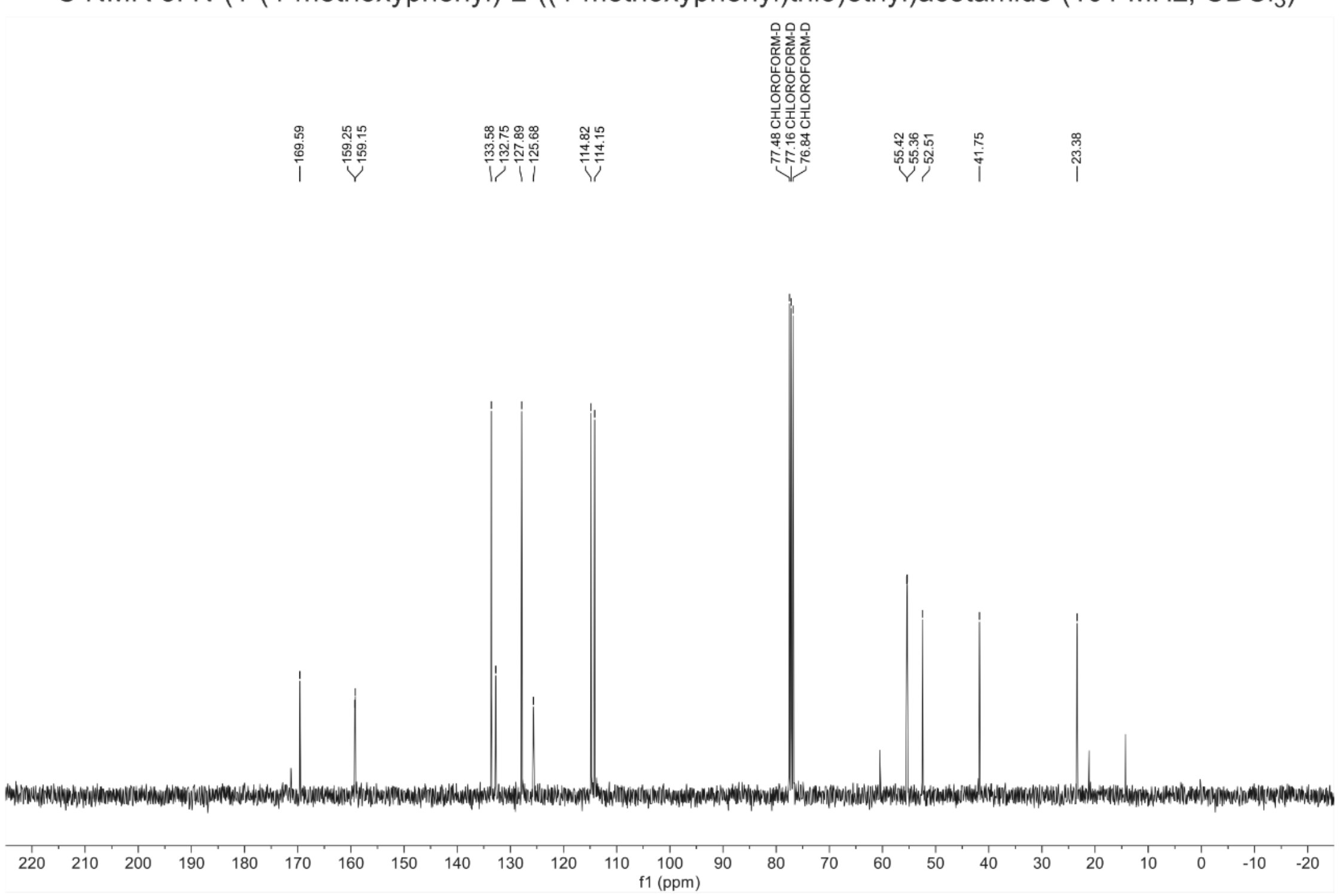

^{1}H NMR of N-(1-(4-methoxyphenyl)-1-((4-methoxyphenyl)thio)ethyl)acetamide (400 MHz, $CDCl_3$)

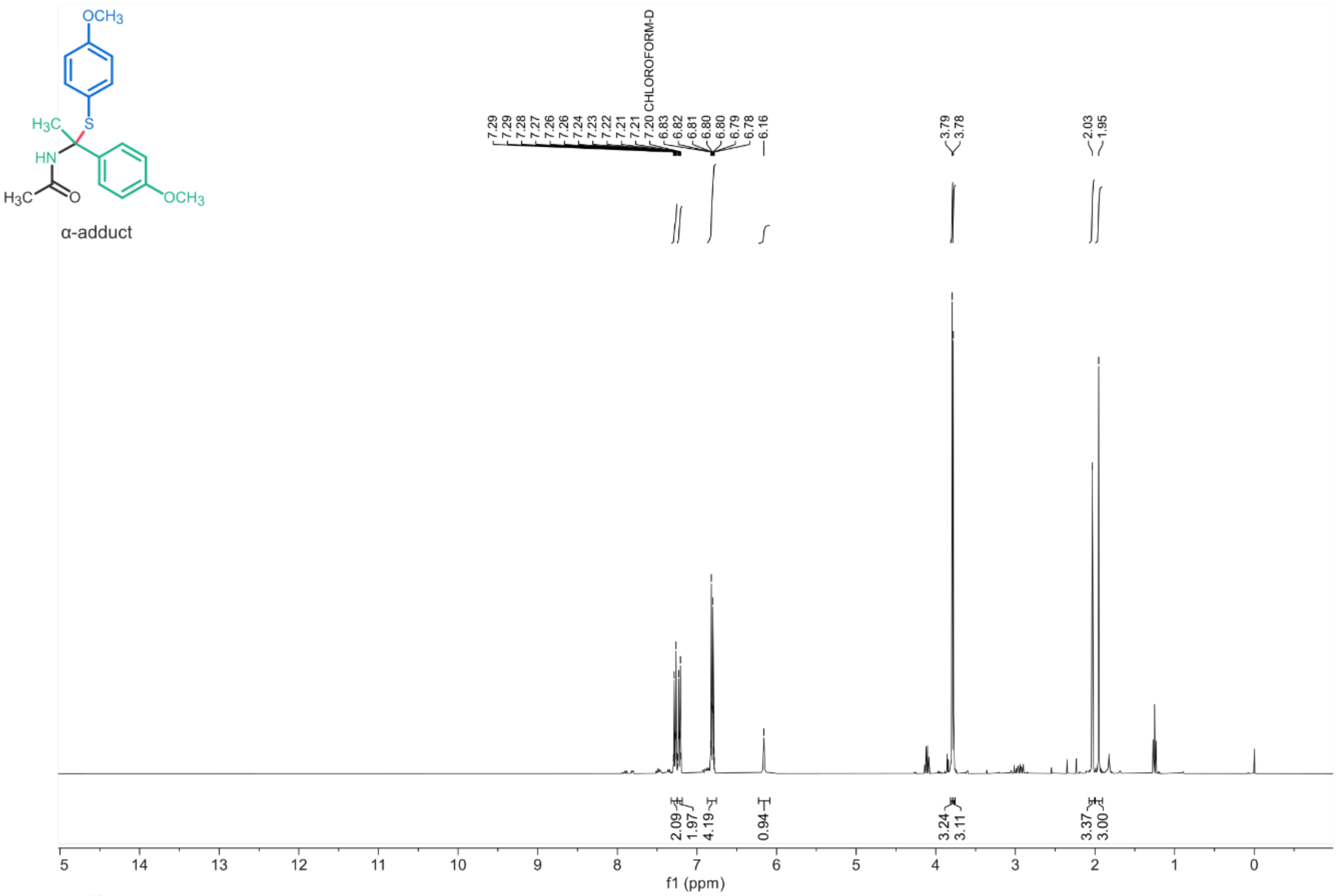

^{13}C NMR of N-(1-(4-methoxyphenyl)-1-((4-methoxyphenyl)thio)ethyl)acetamide (101 MHz, $CDCl_3$)

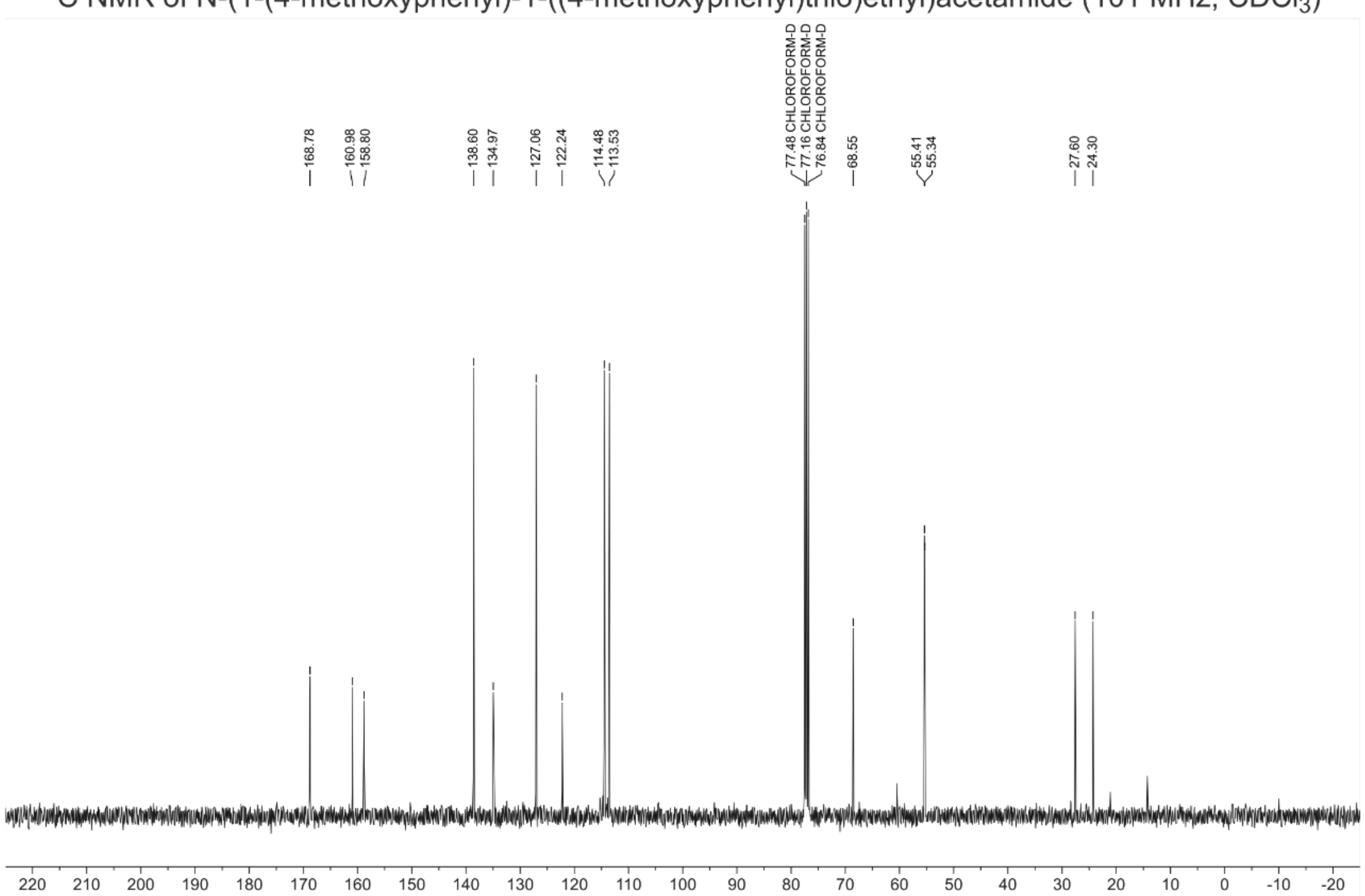

5.2. HPLC/MS Peptide characterization

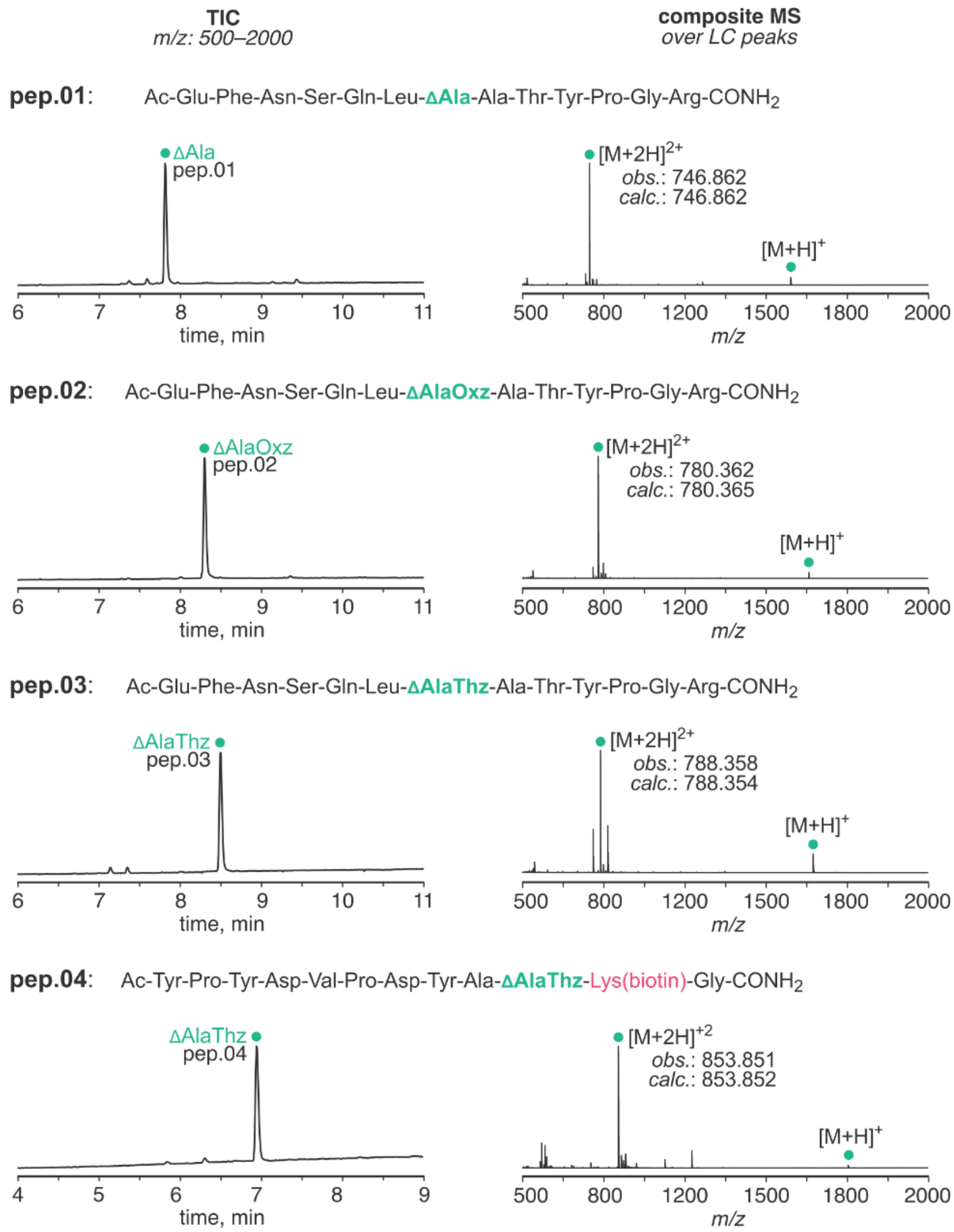

Supplement: Supplementary file 1 [file CB-007-D6CB00161K-s001.pdf]
